# Supplementary material for: Loss of function mutations in GEMIN5 cause a neurodevelopmental disorder
Source: Nat Commun. 2021 May 7;12:2558. doi: 10.1038/s41467-021-22627-w (PMC8105379; doi:10.1038/s41467-021-22627-w)
Supplement: Supplementary file 1 — Supplementary Information [file 41467_2021_22627_MOESM1_ESM.pdf]

Supplementary Figure 1

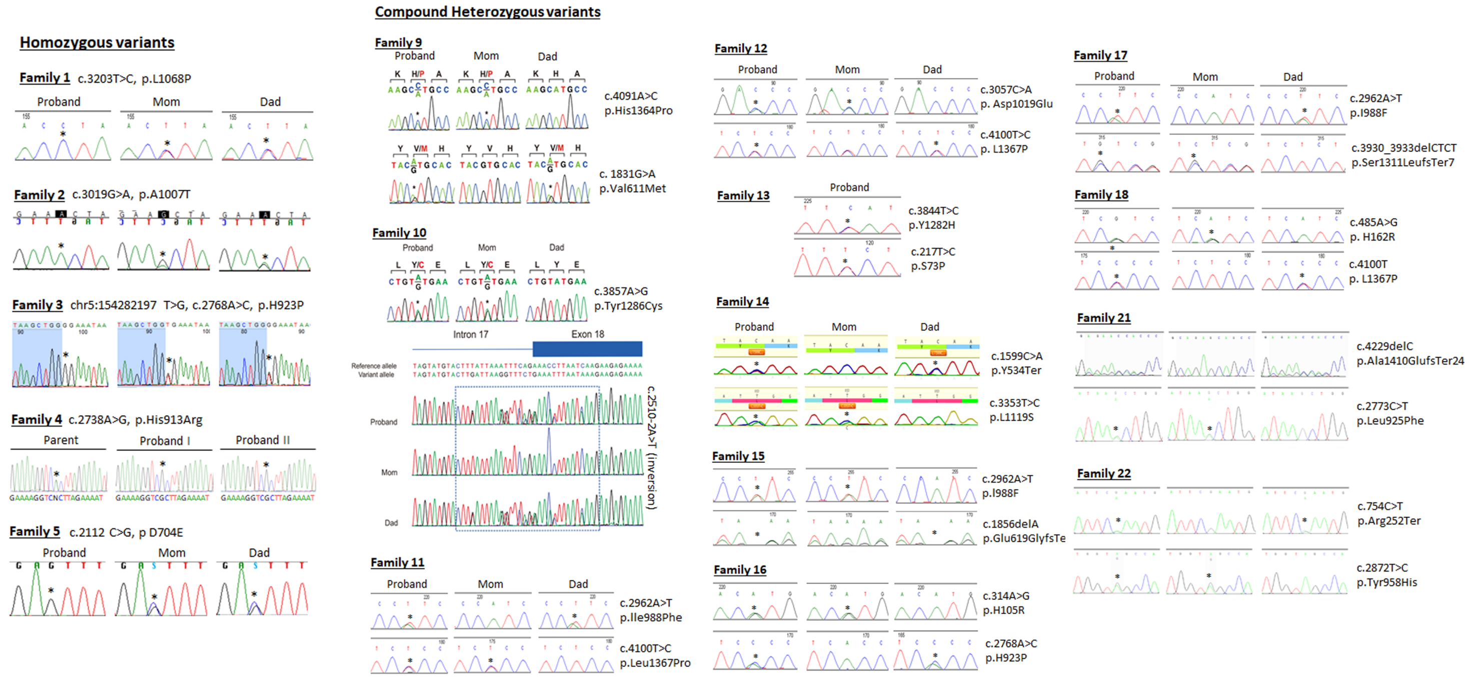

Supplementary Figure 1: Identification of different GEMIN5 variants in various patients and unaffected individuals by exome sequencing. Chromatograms of Sanger confirmation showing the position of compound heterozygous GEMIN5 variants in the patients (proband) as well as parents from the cohort of families identified with common neurological symptoms such as ataxia, hypotonia, and cerebellar atrophy.

Supplementary Figure 2

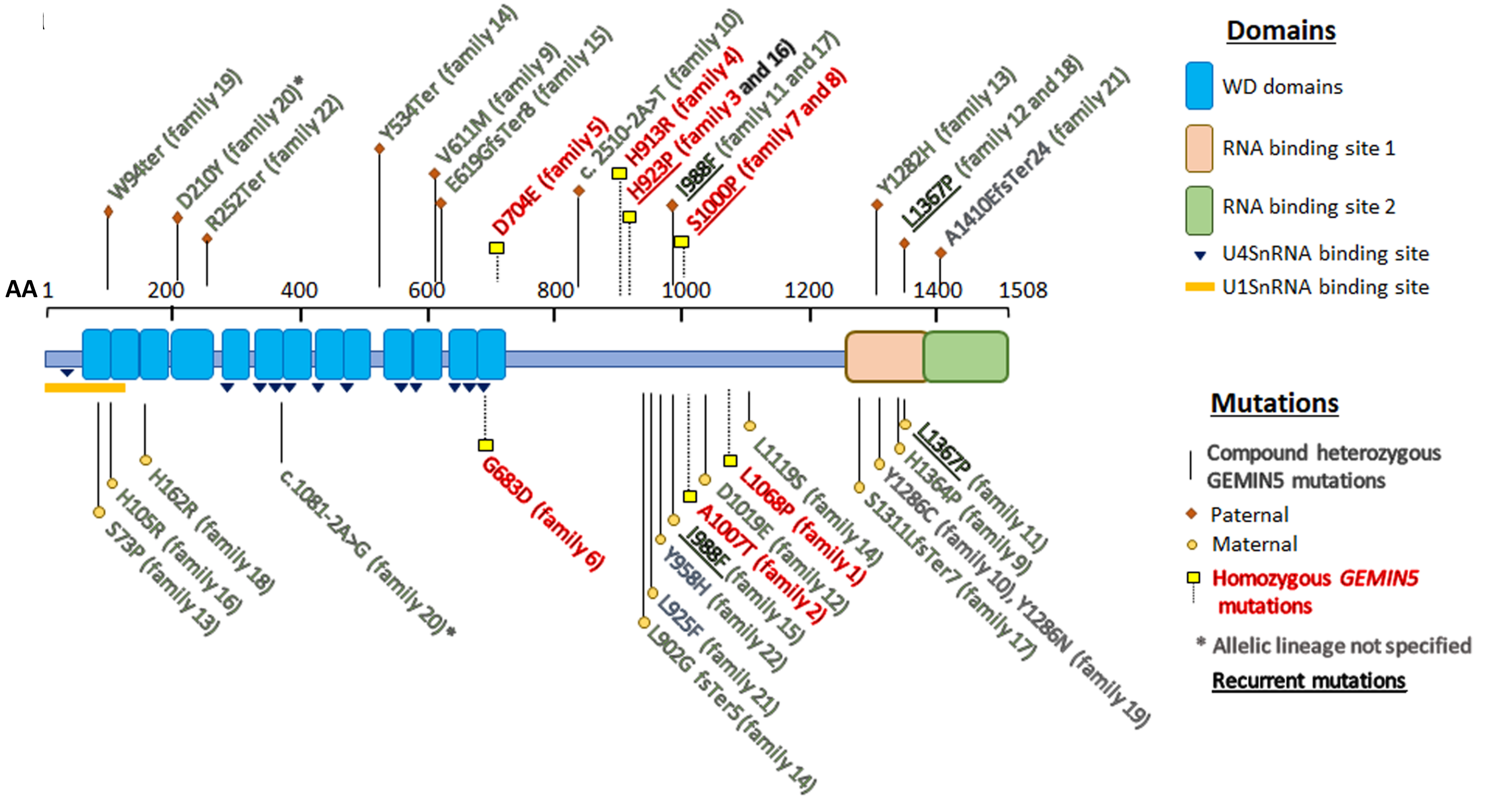

Supplementary Figure 2: Schematic layout of GEMIN5 protein showing the location of the GEMIN5 variants described in this study.

Supplementary Figure 3

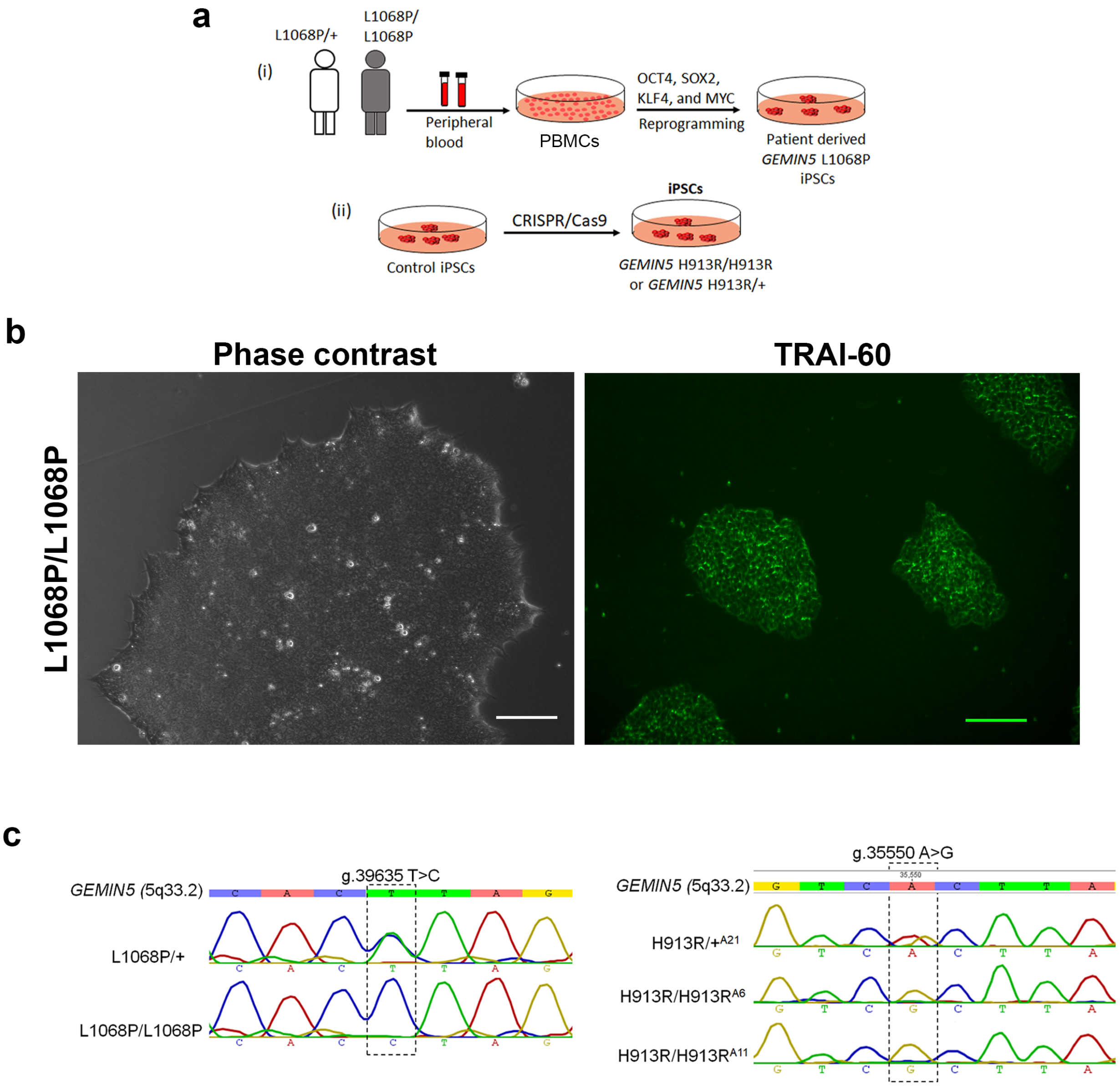

Supplementary Figure 3: Immunofluorescence validation of GEMIN5 L1068P iPSC lines. a, Flowchart depicting the generation of mono- and bi-allelic (L)Leu1068Pro(P) or (H)His913Arg(R) IPSCs (i) from peripheral blood (PB) of patients and unaffected individuals, and (ii) by CRISPR/Cas9 approach. **b**, Phase contrast and immunofluorescent images showing an iPSC cells colony derived from the PMBCs of L1068P-/- carrying individuals. The purity of iPS cells was determined by pluripotency marker TRA1-60. All the images were taken at 10X optical magnification (Scale bar=100μm). **c,** Sequence verification of the iPSC clones with His913Arg (A>G) and Leu1068Pro (T>C) mono- and biallelic variants in GEMIN5.

Supplementary Figure 4

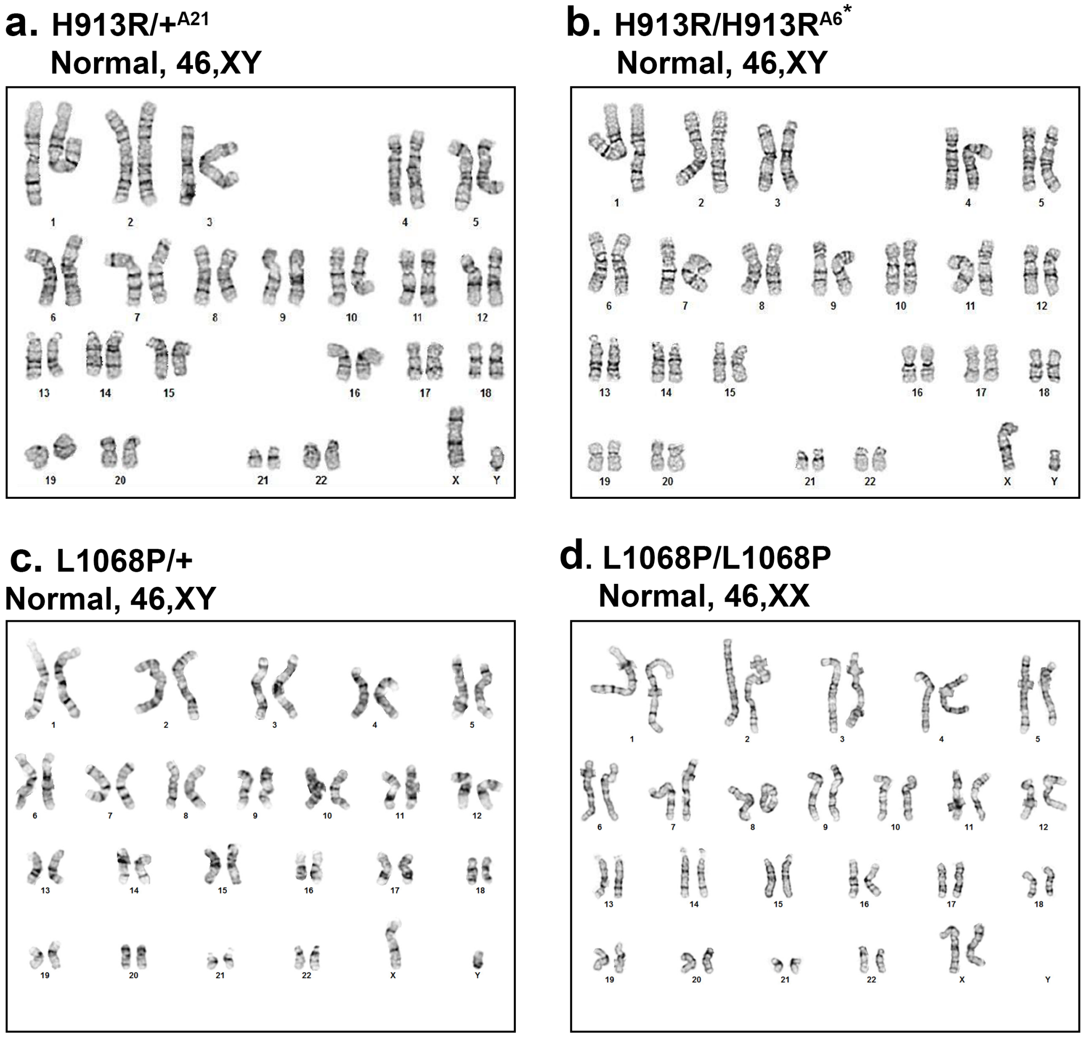

Supplementary Figure 4: G-banding karyotype of His913Arg and Leu1068Pro carrying iPSC lines. a-b, Representative images showing the normal 46, XY karyotype of heterozygous, His913ArgA21 **(a),** and homozygous, His913ArgA6 **(b)** iPSC lines at passage 20 (p20) post- reprogramming by CRISPR/Cas9. c-d, Normal karyotype images of patient-derived iPSC cells carrying heterozygous **(c)** and homozygous **(d)** Leu1068Pro GEMIN5 variants. Cytogenetic analysis was performed on 20 G-banded metaphase cells and all of them showed normal karyotype.

Supplementary Figure 5

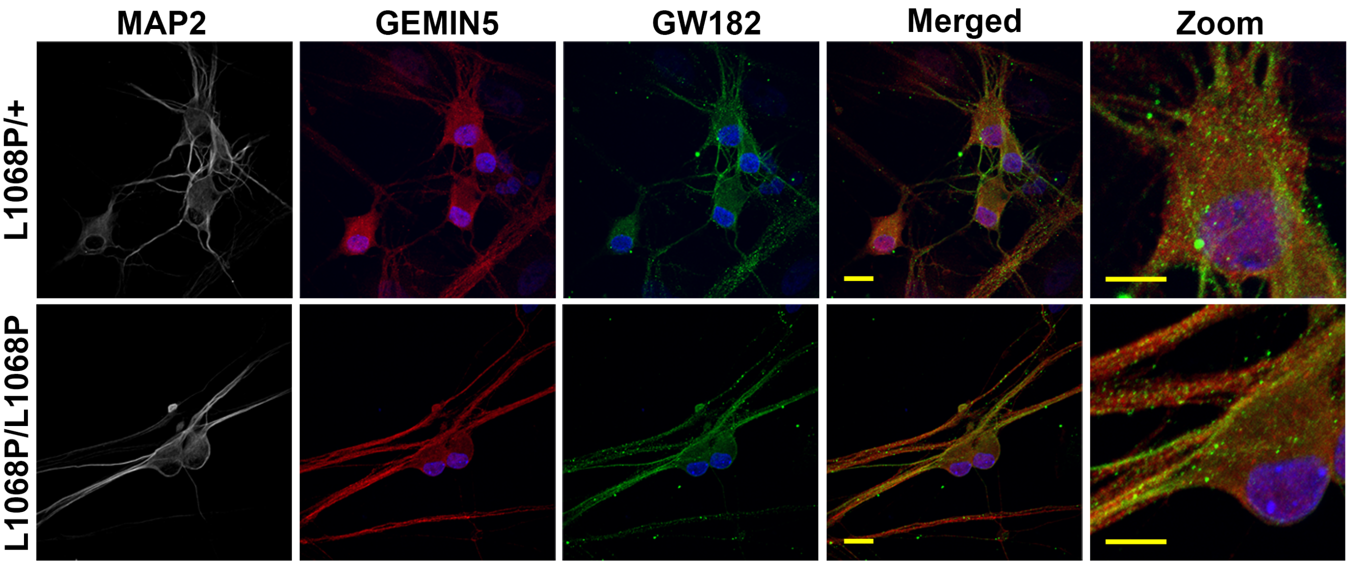

Supplementary Figure 5: Co-localization of GEMIN5 Leu1068Pro variant with GW182. Representative IF images of Leu1068Pro hetero- and homozygous neurons (Leu1068Pro/+ and Leu1068Pro/Leu1068Pro) showing no obvious co-localization of GEMIN5 with p-body marker GW182. MAP2 was used as neuronal marker and the images were captured at 60X (scale bar=10μm).

Supplementary Figure 6

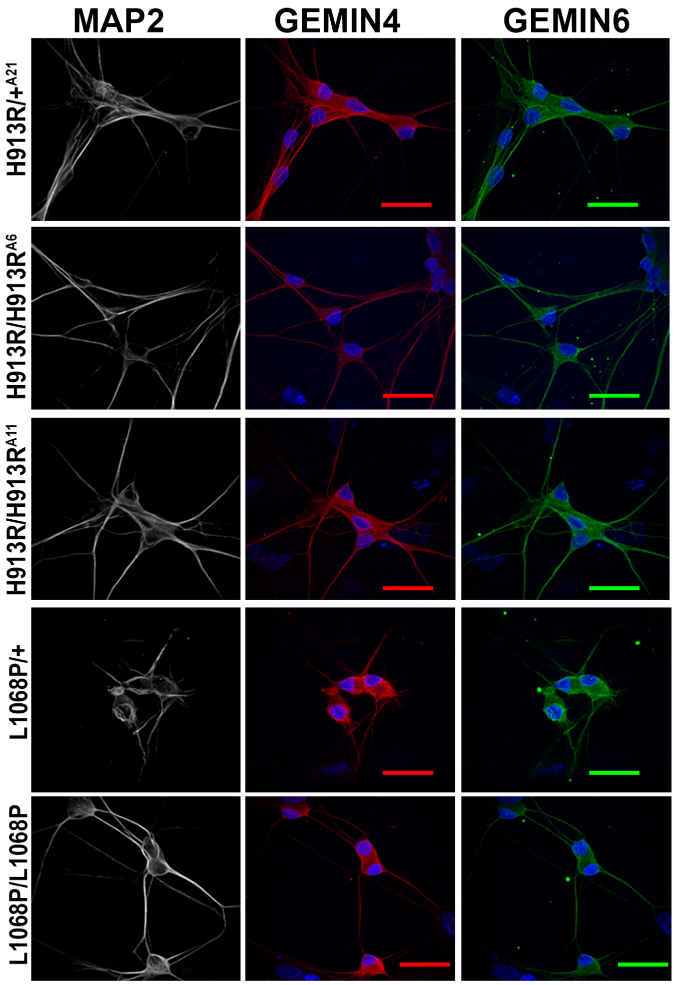

Supplementary Figure 6: Subcellular distribution of GEMIN4 and 6 in neuronal cells expressing GEMIN5 variants. Representative IF images showing no apparent changes in the subcellular expression pattern of GEMIN4 and GEMIN6 between neuronal cells expressing GEMIN5 H913R or L1068P homozygous (H913RA6, H913RA11, and L1068P/L1068P) and heterozygous (H913RA21 and L1068P/+) variants. MAP2 was used as neuronal marker. The images were captured at 60X (scale bar=10μm).

Supplementary Figure 7

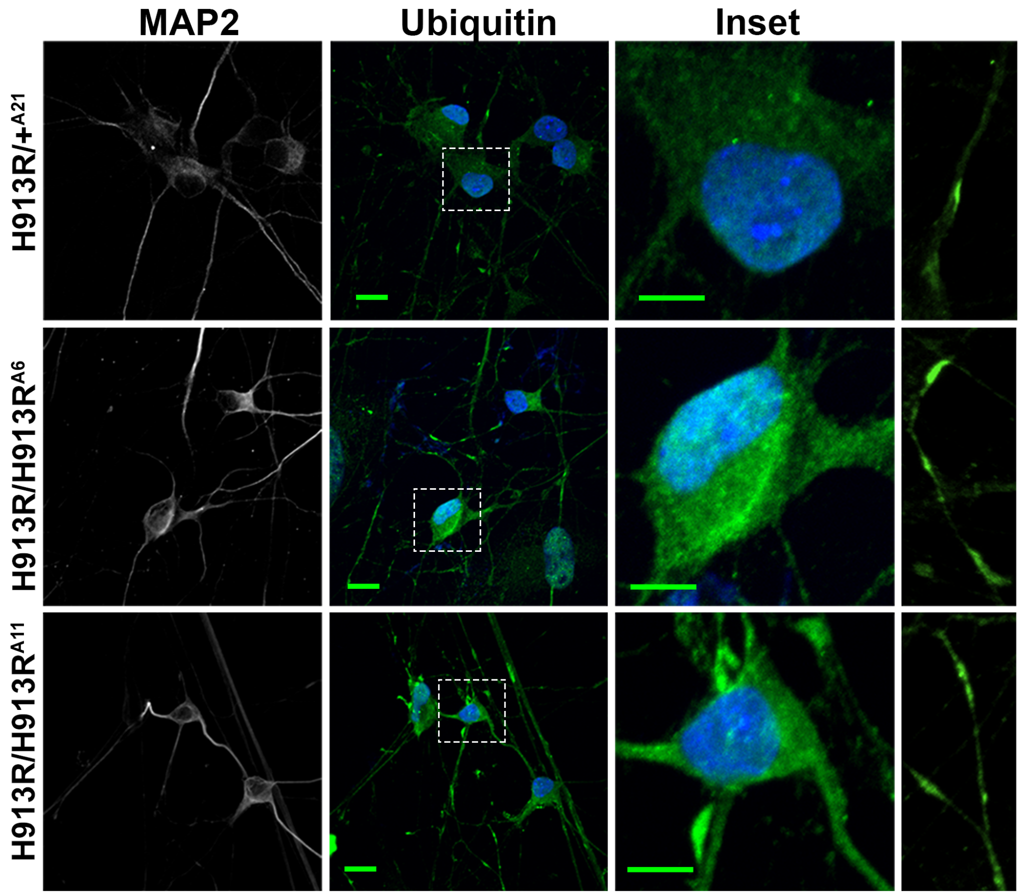

Supplementary Figure 7: Homozygous H913R variants lead to increase ubiquitination. IF images of H913R neurons displaying increase levels of ubiquitin puncta in the cytoplasm and axons of homozygous neurons compared to heterozygous controls (scale bar=10μm).

Supplementary Figure 8

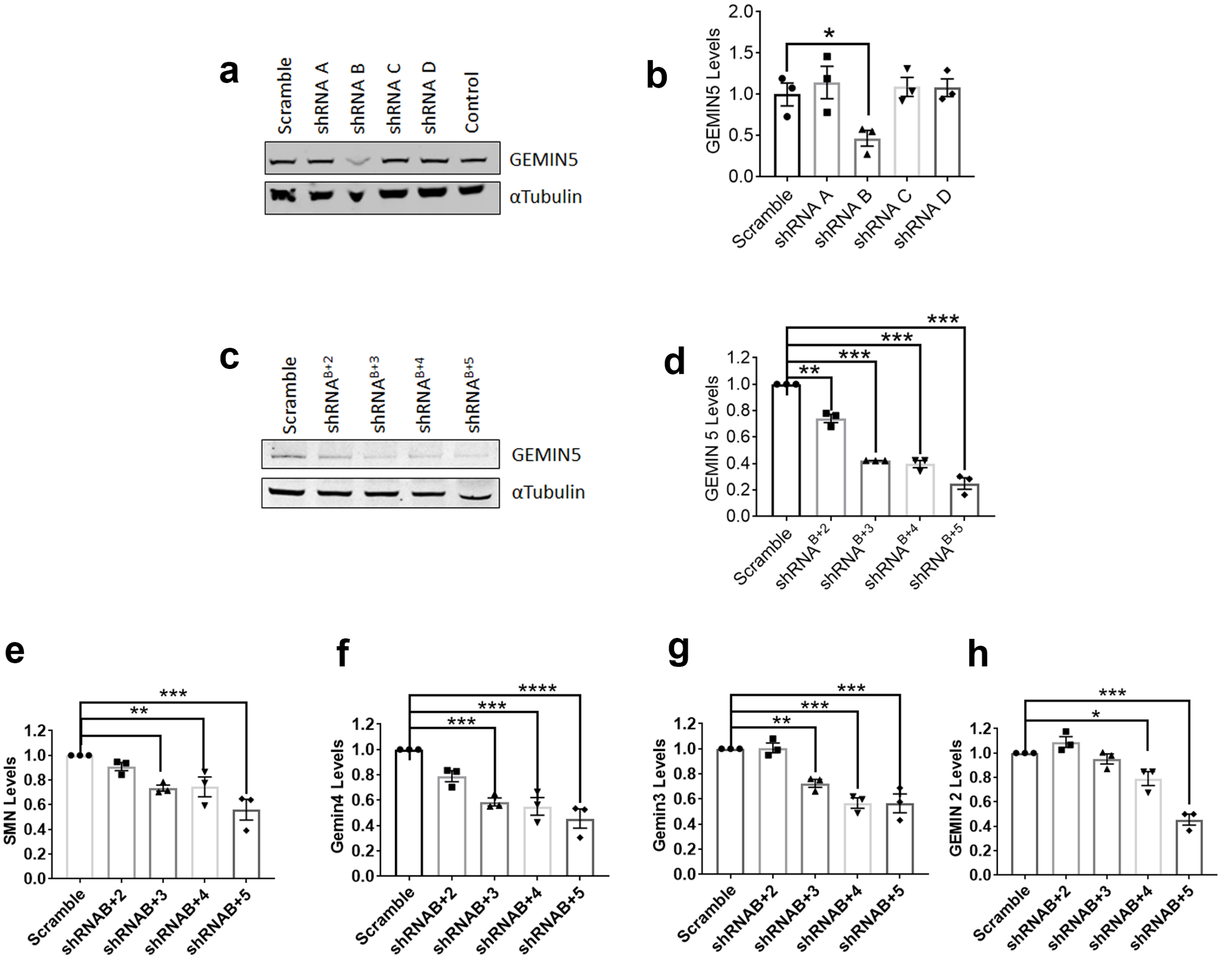

Supplementary Figure 8: Dose dependent effect of loss of GEMIN5 on SMN assembly proteins. a-b, Knockdown validation of different shRNAs from Origene against GEMIN5 in HEK293T cells by WB. Out of four shRNAs, only shRNAb cause significant reduction of GEMIN5 levels **(b)** (one-way ANOVA- Bonferroni test, *n=5*). **c-d,** Representative WB showing the efficiency of shRNA B in knocking down GEMIN5 when combined with four different shRNAs, shRNA 2, 3, 4, and 5 (Dharmacon). As shown in **(d),** shRNA B significantly reduced GEMIN5 levels to 60-80% when used in combination with shRNA 3,4, and 5 (one-way ANOVA- Bonferroni test, n=5). **e-h**, WB showing changes in the protein levels of SMN, GEMIN4, GEMIN3, and GEMIN2 upon different degree of GEMIN5 KD. The percentage decrease in the protein levels of SMN **(f),** GEMIN4 **(g),** GEMIN3 **(h),** and GEMIN2 **(i)** was found to be directly dependent on the amount of GEMIN5 levels in HEK cells (one-way ANOVA- Bonferroni test, *n=5*). Tubulin was used as normalization control. The data represent mean ± SEM. P values (****<0.0001, ***<0.001, **<0.01). Source data are provided as a Source Data file.

Supplementary Figure 9

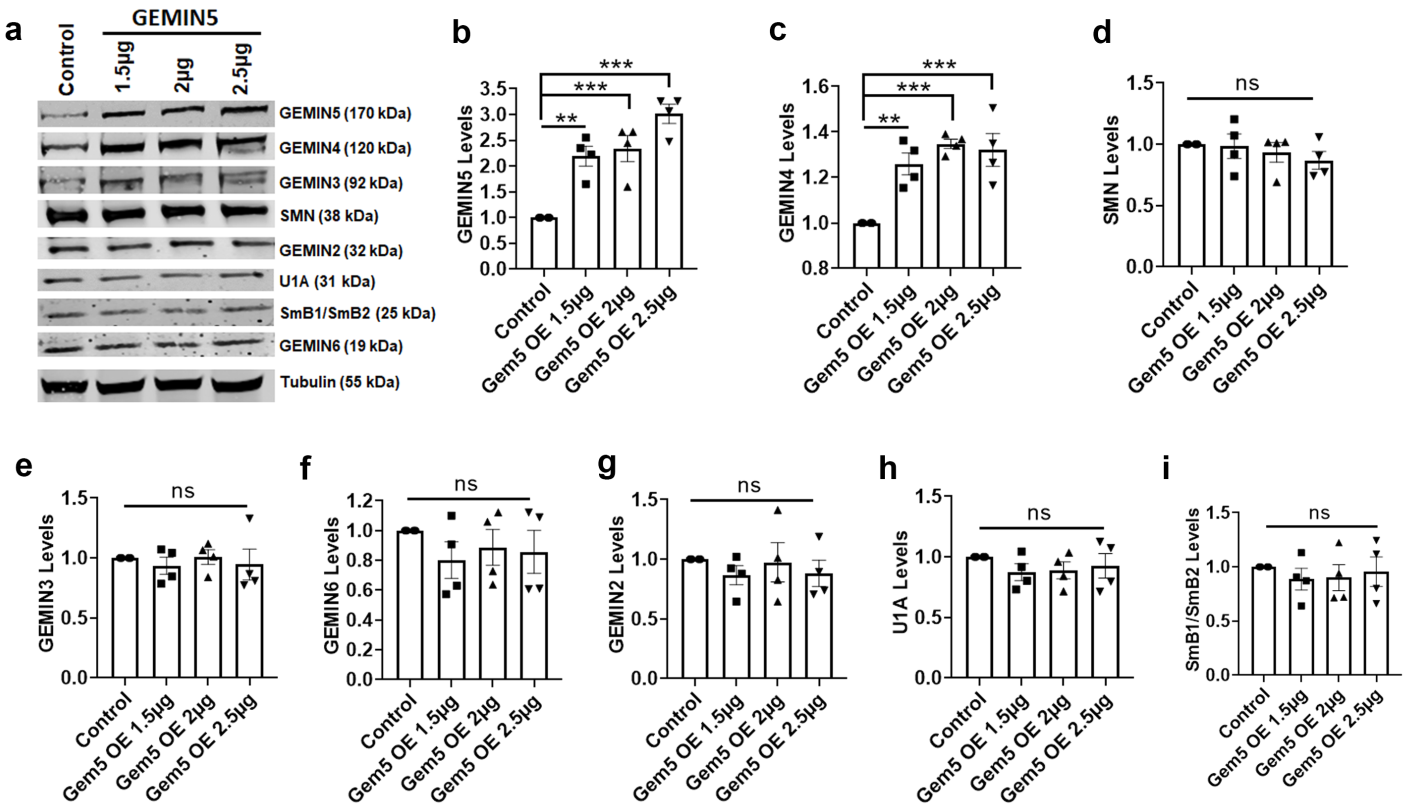

Supplementary Figure 9: Effect of increased levels of GEMIN5 on expression of SMN complex proteins: **a,** Representative immunoblots showing the levels of GEMIN5, SMN, U1A, SmB1/B2, and other GEM proteins after ectopic overexpression of GEMIN5 plasmid construct in HEK293T cells. **b-i,** Quantitative bar graphs showing significant and increase in GEMIN4 **(c)** after dosage-dependent overexpression of GEMIN5 **(b)** and as shown in **(a).** No significant change was observed in the levels of SMN **(d),** GEMIN3 **(e),** GEMIN6 **(f),** GEMIN2 **(g),** U1A **(h),** and SnB1/B2 **(i)** levels by GEMIN5 overexpression. P values (***<0.001, **<0.01, n.s) are of One-way analysis of variance (ANOVA) and post-hoc Bonferroni test. Source data are provided as a Source Data file.

Supplementary Figure 10

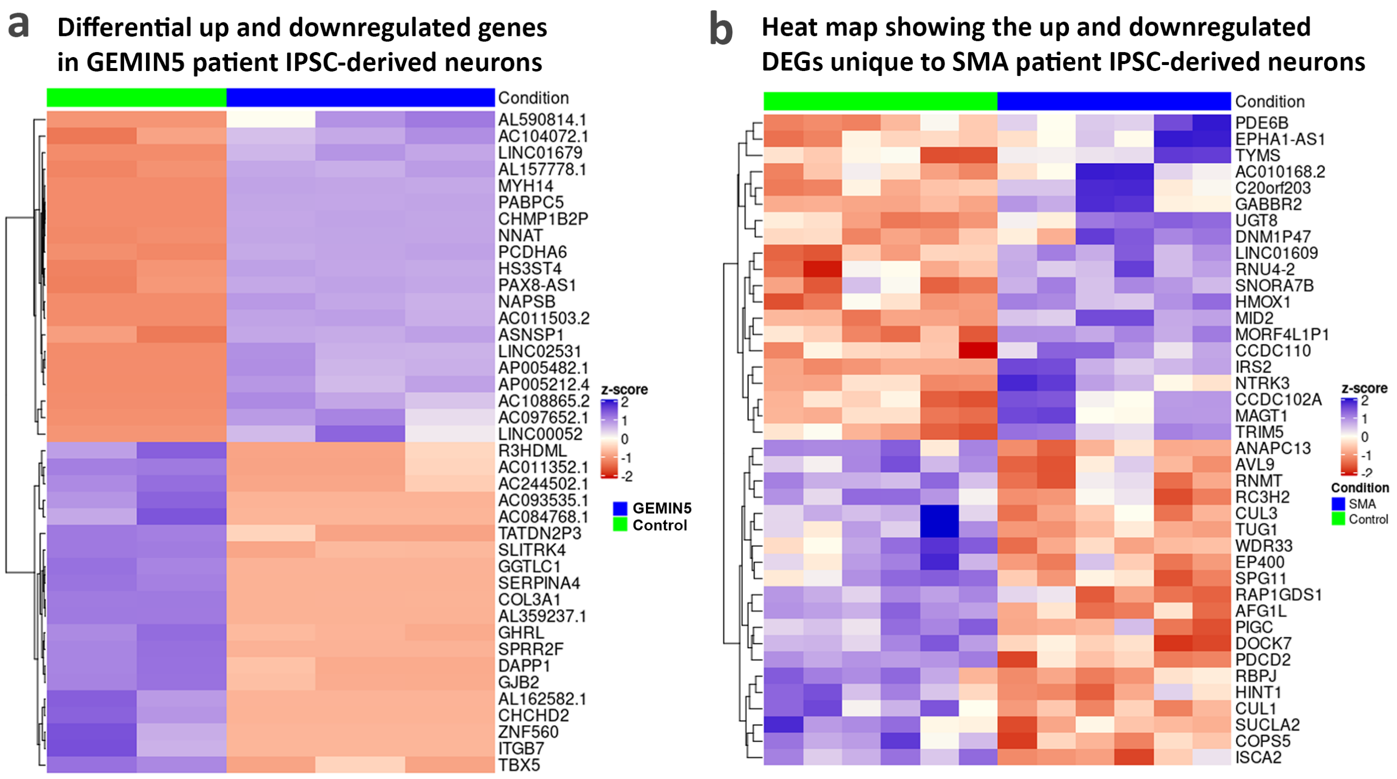

Supplementary Figure 10: a-b, Heat map depicting the hierarchical clustering of top 20 up and downregulated genes which are specific to GEMIN5^{H913R} **(a)** and SMN^{Exon7del} **(b)** as compared to control (Wald test in DESeq2 and multiple test correction by Benjamini and Hochberg's).

Supplementary Figure 11

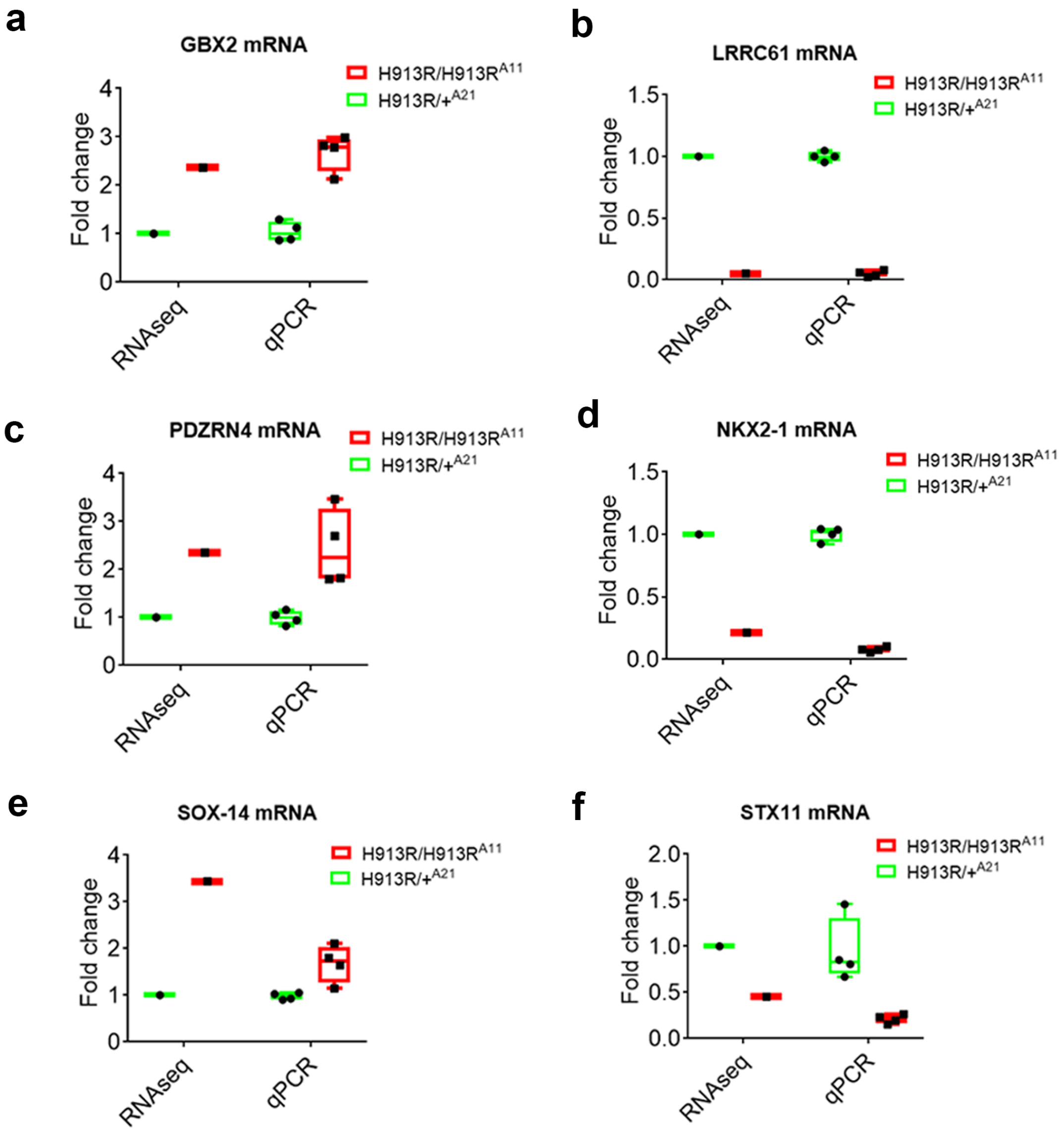

Supplementary Figure 11: a-g, QPCR validation of set of up- and down-regulated genes from H913R-GEMIN5 RNA sequencing. Total RNA isolated from H913R expressing neurons were used to measure the expression levels of transcripts. We found that the expression of GBX2 **(a),** PDZRN4 **(b),** and SOX14 **(c)** were upregulated while STX11 **(d),** NXX.2 **(e),** LRRC1 **(f)** were downregulated in H913R homozygous neurons as compared to heterozygous controls. The data represent mean ± SEM. P values (****<0.0001, ***<0.001, **<0.01) are of two tailed Mann- Whitney U test*, n=4*. Source data are provided as a Source Data file.

Supplementary Figure 12

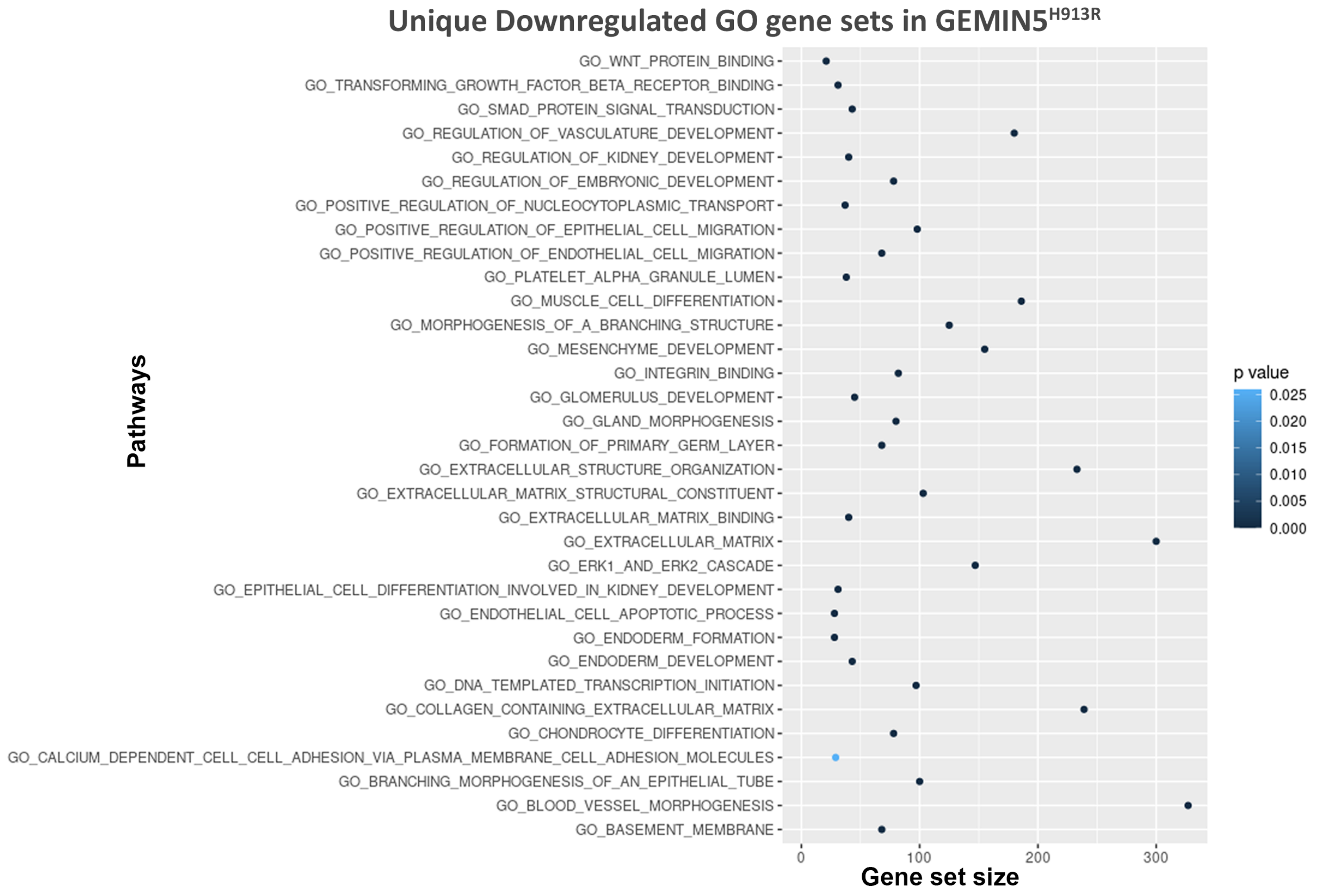

Supplementary Figure 12: Functional characterization of the genes with the MSigDB 'c5 Gene Ontology (GO), Biological Process Ontology (BP) v6.0' downregulated gene sets unique to GEMIN5H913R. The cutoff was set at p-value and FDR value ≤0.05 (Benjamini and Hochberg’s approach). Source data are provided as a Source Data file.

Supplementary Figure 13

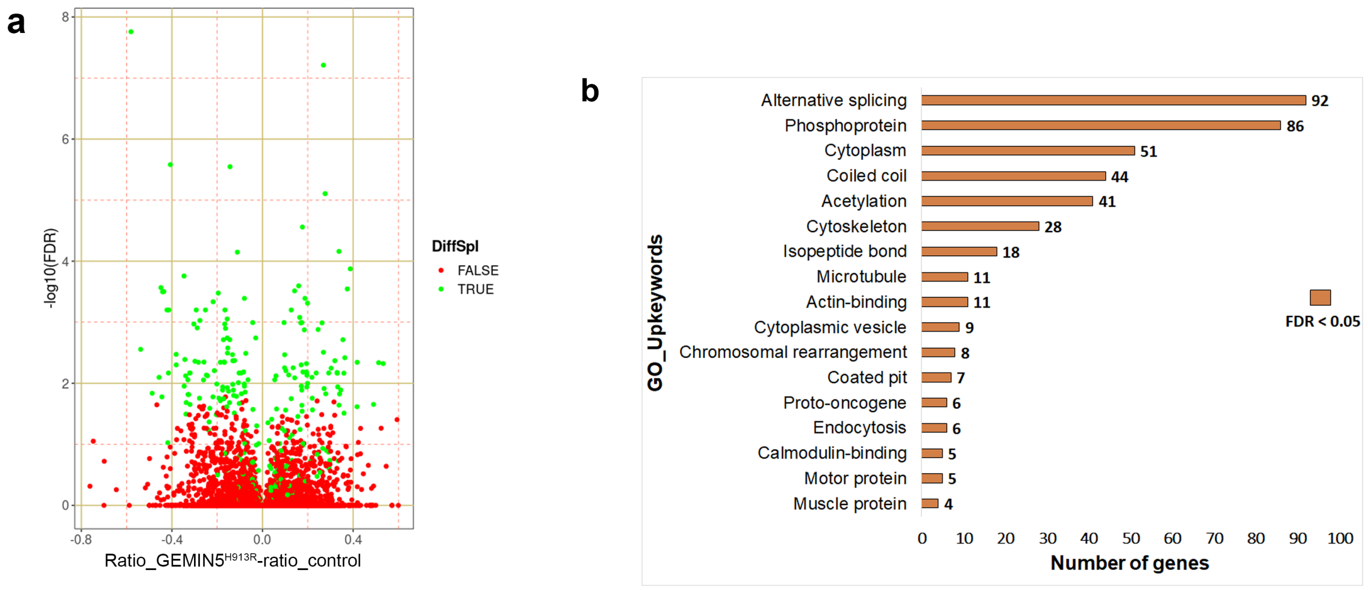

Supplementary Figure 13: a, A volcano plot showing the difference between isoform's relative expression in the contrasted condition on the x-axis in homozygous GEMIN5 H913R vs control. The isoforms are colored according to the differential splicing status of the gene they come from, adjusted at a 5% threshold. **b,** Functional enrichment analysis of differentially spliced genes (DSGs) in GEMIN5H913R neurons compared to controls. The DAVID algorithm was used for the analysis. The x-axis represents gene ontology (GO) annotation for up-keywords pathways with FDR < 0.05. Source data are provided as a Source Data file.

Supplementary table 1: Clinical summary of patients carrying GEMIN5 mutations

**Family**	**1**	**2**		**3**	**4**		**5**	**6**		**7**		**8**		**9**		**10**	**11**	**12**		**13**	**14**	**15**	**16**	**17**		**18**	**19**	**20**	**21**	**22**
**Patient number**	1	2	3	4	5	6	7	8	9	10	11	12	13	14	15	16	17	18	19	20	21	22	23	24	25	26	27	28	29	30
**Gender**	F	F	F	M	M	M	M	M	M	F	F	M	M	M	M	F	M	F	M	M	M	M	F	M	M	F	F	M	M	F
Age of onset Birth **(B)**	6 m	1st y	1st y	B	B	B	11 m	1st	1st	1st y	1st y	2m	2m	<1 y	10 m	6m		7m	2y	B	1y	1 y	1y	<1y	B	B	4m	B	10m	1y
Current Age Year (Y) Deceased **(D)**	7 y	5 y	3 y	D	D	D	?	9y	7y	9y	4y	10y	2y	?	?	4 y	15 y	8y	6y	7y	31y		7y	29y	27 y	18 m	31 m	5y	3y	4y
**Development**																														
Delayed? **Yes: Y** **No: N**	Y	Y	Y	Y	Y	Y	Y	Y	Y	Y	Y	Y	Y	Y	Y	Y	Y	Y	Y	Y	Y	Y	Y	Y	Y	Y	Y	Y	Y	Y
Regression Y/N	N	N	N	N	N	N	N	N	N	N	N	N	N	N	N	N	N	N	N	N	N	N	N	N	N	N	N	N	N	N
Motor delay Y/N	Y	Y	Y	Y	Y	Y	Y	Y	Y	Y	Y	Y	Y	Y	Y	Y	Y	Y	Y	Y	Y	Y	Y	Y	Y	Y	Y	Y	Y	Y
Speech delay Y/N/NA	Y	Y	Y	Y	NA	NA	Y	Y	Y	Y	Y	Y	Y	Y	Y	Y	Y	Y	Y	Y	Y		Y	Y	Y	N	Y	Y	Y	Y
Cognitive delay Y/N/NA NA: Not applicable	Y	Y	Y	Y	NA	NA	Y	Y	Y	Y	Y	Y	Y	Y	Y	Y	Y	Y	Y	Y	Y		Y	Y	Y	N	Y	Y	Y	Y
**Neurological findings**																														
Ataxia Not walking **(NW)**	Y	Y	NW	NW	N/A	NA	Y	Y	Y	Y	Y	NW	NW	Y	NA	Y	Y	Y	Y	Y	Y	NW	Y	Y	Y	NW	NW	Y	NW	Y
Appendicular Hypertonia Y/N	N	Y	Y	N	N	N	N	Y	Y	Y	Y	N	N	N	N	N		Y	Y	Y	Y	N	N	Y	Y	N	Y	N	N	N
Central Hypotonia Y/N	Y	Y	Y	Y	Y	Y	Y	Y	Y			Y	Y	Y	Y	Y	Y	Y	Y	Y	Y	Y	Y	N	N	Y	Y	Y	Y	N
Deep tendon reflexes Normal (N) Brisk **(B)** **Absent (A)**	N	N	B	A	A	A	?	B	B	B	B	N	N	?	?	B		B	B	B	B	B	?	B	B	A	B	B	N	N
**Neurological evaluation**																														
Cerebellar atrophy (MRI) Yes (Y)/ No (N)	Y	Y	Y	Y	Y	Y	Y	Y	Y	Y	Y	Y	Y	Y	Y	Y	Y	Y	Y	Y	Y	Y	Y	Y	Y	Y	Y	Y	Y	Y
**Clinical Course**																														
Static **(S)**/ Progressive **(P)**	S	S	S	P	N/A	N/A	S	S	S	S	S	S	S				P	S	S	P	S			P	P	S	S	S	S	P
Genetic variant	L1068P	A1007T		H923P	H913R		D704E	G683D		S1000P		S1000P		V611M/ H1364P		2510-2A>T/Y1286C	I988F/L1367P	L1367P/ D1019E		Y1282H/S73P	L1119S/Y534X	I988F/ E619GfsTer8	H105R/ H923P	I988F/ Ser1311 LeufsTer7		H162R/ L1367P	W94Ter/Y1286Asn	c.1081-2A>G/ D210Y	A1410EfsTer24/ p.L925F	R252Ter/Y958H

Supplementary table 2: List of patients with GEMIN5 mutations examined by EMG/NCV

Patient	EMG/ NCV	Muscle Biopsy
4	Electrophysiological testing: sensory nerve conductions showed normal latency, amplitude, and velocity. The motor nerve revealed a significant reduction amplitude CMAPs and normal latency and velocities in upper limbs, in lower limbs the motor nerves were unexcitable. Electromyography showed in all muscles explored a severe neurogenic pattern with abundant spontaneous activity at rest (fibrillations and positive waves were present).	
5	Pathological spontaneous activity and slight neurogenic changes of action potentials. Motor nerve conduction was markedly reduced. Sensory nerve potentials could not be elicited.	Muscle biopsy was notable for large group atrophy and sural nerve displayed loss of large, myelinated axons, with no evidence of demyelinating neuropathy.
7	Lower extremities with increased duration and amplitude of motor unit potentials (MUAPs) with preserved recruitment	
8	Neurogenic pattern, with reduction of amplitude of motor nerves rather than sensory, but mild delayed latencies in upper limbs	
9	Neurogenic pattern, with reduction of amplitude of motor nerves rather than sensory, but normal latencies in upper limbs	
10	Normal	
11	Normal	
12	Neuropathic, fibrillation potentials, denervation, and increased amplitude suggestive of anterior horn cells affection	
13	Neuropathic, fibrillation potentials, denervation, and increased amplitude suggestive of anterior horn cells affection	
17	Consistent with a long-standing disorder of motor neurons	
18	Normal	
22	Myopathic process with large motor units from tibialis anterior	
25	The findings support a possible chronic involvement of muscle as part of this multisystemic process. Chronic loss of anterior horn cells could also result in a similar pattern, but there is no abnormal spontaneous activity to support active ongoing denervation, if this were the case.<br>EMG/NCV10 years later: The electrophysiologic findings are most suggestive of a moderate chronic and ongoing motor neuropathy or motor neuronopathy. An incidental note is made of a median neuropathy at the right wrist (as in carpal tunnel syndrome)	

26	Left median, peroneal, and tibial motor responses were absent. Left sural sensory nerve action potential (SNAP) was not obtainable. Needle EMG of selected muscles of the left upper and lower extremities showed abnormal spontaneous activity with fibrillation potentials and fasciculations. During voluntary activation no motor unit action potentials were seen in the lower extremities. In the bicep’s recruitment was reduced with large polyphasic units. Interpretation: This is an abnormal study. There is electrodiagnostic evidence for severe neuropathic disorder.	
29	Normal	
30	Normal	

Supplementary Table 3: Allelic frequencies of the GEMIN5 variants identified in our study

Family	*GEMIN5* Variant (cDNA) (NM_015465.5)	*GEMIN5* Variant (protein) (NP_056280.2)	Allele frequency (heterozygous)	Number of homozygous
1	c.3203T>C	Leu1068Pro	3.98e-5	0
2	c.3019G>A	Ala1007Thr	0	0
3	c.2768A>C	His923Pro	0	0
4	c.2738A>G	His913Arg	0	0
5	c.2112C>G	Asp704Glu	0	0
6	c.2049C>T	Gly683Asp	0	0
7	c.2995T>C	Ser1000Pro	0	0
8	c.2995T>C	Ser1000Pro	0	0
9	c.1831G>A<br>c.4091A>C	Val611Met,<br>His1364Pro	8.07e-6<br>0	0<br>0
10	c.3857A>G<br>2510-2A>T (inversion)	Tyr1286Cys,<br>2510-2A>T (inversion)	3.99e-6<br>0	0<br>0
11	c.2962A>T<br>c.4100T>C	Ile988Phe<br>Leu1367Pro	9.15e-5<br>3.89e-5	0<br>0
12	c.4100T>C<br>c.3057C>A	Leu1367Pro<br>Asp1019Glu	3.89e-5<br>0	0<br>0
13	c.3844T>C<br>c.217T>C	Tyr1282His<br>Ser73Pro	4.01e-6<br>0	0<br>0
14	c.3356T>C<br>c.1602C>A	Leu1119Ser<br>Tyr534Ter	0<br>0	0<br>0
15	c.2962A>T<br>c.1856delA	Ile988Phe<br>Glu619GlyfsTer8	9.15e-5<br>1.23e-5	0<br>0
16	c.314A>G<br>c.2768A>C	His105Arg<br>His923Pro	0<br>0	0<br>0
17	c.2962A>T<br>c.3930_3933delCTCT	Ile988Phe<br>Ser1311LeufsTer7	9.15e-5<br>3.98e-6	0<br>0
18	c.485A>G<br>c.4100T>C	His162Arg<br>Leu1367Pro	0<br>3.89e-5	0<br>0
19	c.282G>A<br>c.3856T>A	Trp94ter<br>Tyr1286Asn	0<br>0	0<br>0
20	c.1081-2A>G (Splice acceptor variant),<br>c.628G>T	1081-2A>G (splice acceptor)<br>Asp210Tyr	0<br>0	0<br>0
21	c.4229delC<br>c.2773C>T	Ala1410GlufsTer24<br>Leu925Phe	0<br>0	0<br>0
22	c.754C>T<br>c.2872T>C	Arg252Ter<br>Tyr958His	0<br>3.98e-6	0<br>0

Supplementary Table 4: List of various *in-silico* prediction tools measuring the severity of *GEMIN5* variants found in affected families.

GEMIN5 Variants	Prediction tools					
	**PholyPhen-2**	**PROVEAN**	**SNAP2**	**mu PRO**	**PhD SNP**	**SIFT**
**p.(Leu1068Pro)**	**Damaging**	**Deleterious (-4.922)**	**Pathogenic**	**Decreased Stability DDG=-2.21579**	**Disease Causing**	**Disease Causing**
**p.(Ala1007Thr)**	**Damaging**	**Deleterious (-3.200)**	**Neutral**	**Decreased Stability DDG=-0.97637**	**Neutral**	**Disease Causing**
**p.(His923Pro)**	**Damaging**	**Deleterious (-2.189)**	**Pathogenic**	**Decreased Stability DDG=-0.77411**	**Disease Causing**	**Disease Causing**
**p.(His913Arg)**	**Damaging**	**Deleterious (-4.778)**	**Pathogenic**	**Decreased Stability DDG=-0.57662**	**Disease Causing**	**Disease Causing**
**p.(Asp704Glu)**	**Damaging**	**Deleterious (-3.489)**	**Pathogenic**	**Decreased Stability DDG=-0.57662**	**Disease Causing**	**Disease Causing**
**p.(Gly683Asp)**	**Damaging**	**Deleterious (-5.883)**	**Neutral**	**Decreased Stability DDG=-0.65471**	**Disease Causing**	**Neutral**
**p.(Ser1000Pro)**	**Light**	**Neutral (-2.483)**	**Neutral**	**Decreased Stability DDG=-1.2372**	**Disease Causing**	**Disease Causing**

Supplementary Table 5: List of primers used

Gene	IDT Assay ID/ Sequence
Gemin5	Primer 1: GGCACTGAAGAGGGTGTATTT<br>Primer2: GGCACTGAAGAGGGTGTATTT<br>Probe: /56-FAM/TGGAGGTGA/ZEN/ACTGTTGCAATGGGA/3IABkFQ/
Gemin4	Hs.PT.58.292685
Gemin2	Hs.PT.58.40563922
Gemin6	Hs.PT.58.25330227
Gemin3	Hs.PT.58.19653938
SMN	Primer1: TGGTGGTCCAGAAGGAAATG<br>Primer2: CCAGGAAAGCCAGGTCTAAA<br>Probe: /56-FAM/CCACTTACT/ZEN/ATCATGCTGGCTGCCT/3IABkFQ/
NKX2-1	Hs.PT.58.2461055
PDZRN4	Hs.PT.58.26664645
GBX2	Hs.PT.58.803756
LRRC61	Hs.PT.56a.39239322
SOX14	Hs.PT.58.27313496
STX11	Hs.PT.58.4557357
Rigor mortis	Primer1: GCTCCTTCTGGGTAAGGTAAAG<br>Primer2: GTGCCAAAGAATGCCCAAAG<br>Probe: /56-FAM/AGTTTCACA/ZEN/CATAGCCGCACCTGT/3IABkFQ/
DmTubulin	Primer1: CCTCGAAATCGTAGCTCTACAC<br>Primer2: ACCAGCCTGACCAACATG<br>Probe: /56-FAM/TCACACGCG/ZEN/ACAAGGAAAATTCACAGA/3IABkFQ/
GAPDH	Hs.PT.39a.22214836
