# Supplementary material for: Loss of function mutations in GEMIN5 cause a neurodevelopmental disorder
Source: Nat Commun. 2021 May 7;12:2558. doi: 10.1038/s41467-021-22627-w (PMC8105379; doi:10.1038/s41467-021-22627-w)
Supplement: Supplementary file 3 — Description of Additional Supplementary Files [file 41467_2021_22627_MOESM3_ESM.pdf]

Description of Additional Supplementary Files

Supplementary Data 1: Clinical Summary
