# Supplementary material for: Loss of function mutations in GEMIN5 cause a neurodevelopmental disorder
Source: Nat Commun. 2021 May 7;12:2558. doi: 10.1038/s41467-021-22627-w (PMC8105379; doi:10.1038/s41467-021-22627-w)
Supplement: Supplementary file 4 — Supplementary data 1 [file 41467_2021_22627_MOESM4_ESM.docx]

**Clinical summary:**

Pedigrees for the family 1-8 are showing in the figure 1.

**Family 1:** c.3203T>C; p.Leu1068Pro

A three-year-old girl, born to non-consanguineous parents of Caucasian origin, was evaluated for history of motor predominant developmental delay (first observed at age 6 months), tremor, and unsteady gait. She also had speech and mild cognitive delay. A pertinent neurological exam showed central hypotonia and ataxia with easily elicitable reflexes. MRI of the brain showed cerebellar atrophy. An EMG was not performed. She had extensive metabolic work up with negative results. Trio whole exome sequencing (WES) revealed a novel homozygous variant in the GEMIN5 gene, p. (Leu1068Pro). The asymptomatic parents and three sisters were found to be heterozygous for the same variant. She was seven years old at last follow up and has remained clinically stable with stable MRI on repeat brain imaging.

**Table. Clinical information and supplementary investigations**

|  | Patient 1 |
| --- | --- |
| Gender | Female |
| Age of symptom onset | 6 months |
| Age at last observation | 7 years |
| **Development** | |
| Delayed? Y/N | Y |
| Regression Y/N | N |
| Motor delay | Y |
| Speech delay | Y |
| Cognitive delay | Mild |
| **Neurological findings** | |
| Ataxia | Y |
| Hypertonia | N |
| Central Hypotonia | Y |
| Deep tendon reflexes | 2+ bilaterally |
| Gait | Wide based, ataxic |
| Tremor/ abnormal movements | Y |
| **Neurological evaluation** | |
| MRI | Cerebellar atrophy |
| EMG | Not done |
| **Clinical Course** | |
| Static/ Progressive | Static |
| **Miscellaneous** | Tremor |
| **Genetic Testing** | c.3203T>C; p.(Leu1068Pro) Homozygous |

**Family 2:**  c.3019G>A; p.Ala1007Thr

Two siblings, a three-year old girl and a five-year old girl born to consanguineous parents of Algerian descent, were evaluated for developmental delay. The delays were observed before the age of one year. The five-year-old girl is currently talking in sentences but is dysarthric. She walks with support but with an unsteady ataxic gait. She was assessed to have an IQ between 70-80 and is toilet trained. Neurological exam showed a mild central hypotonia, nystagmus, ataxia, appendicular hypertonia, along with easily elicitable reflexes. Brain MRI revealed cerebellar atrophy, along with mild FLAIR changes in the posterior occipital white matter. The sibling, now 3 years old, recently started walking with support and can talk in two-word sentences with a dysarthric speech and is estimated to have mild-moderate cognitive delay. Her neurological exam was notable for mild central hypotonia, nystagmus, appendicular hypertonia and brisk reflexes. Her brain MRI also showed cerebellar atrophy with minimal periventricular FLAIR changes. Their clinical course has been static. Exome testing (WES) revealed novel homozygous variants, p.(Ala1007Thr), in the GEMIN5 gene.

**Table. Clinical information and supplementary investigations**

|  | Patient 2 | Patient 3 |
| --- | --- | --- |
| Gender | Female | Female |
| Age of symptom onset | 1 st year of life | 1^st^ year of life |
| Age at last observation | 4 year and 5 months | 3 years |
| **Development** | | |
| Delayed? Y/N | Y delayed from the start | Y delayed from the start |
| Regression Y/N | N | N |
| Motor delay | Sat: 1 year and 6 months  Stand with support recently  Cannot walk alone, walk only few steps with support | Can sit only after 2 years  Cannot walk till now |
| Speech delay | 1^st^ spoke :9 months now can talk sentence | Mild language delay, only words |
| Cognitive delay | Moderate cognitive delay | Mild cognitive delay, she has good eye follow |
| **Neurological findings** | | |
| Ataxia | Y | Cannot assessed |
| Hypertonia | Y | Y |
| Central Hypotonia | Y | Y |
| Deep tendon reflexes | Present | Brisk |
| Gait | Unsteady | Cannot walk |
| **Neurological evaluation** | | |
| MRI | Cerebellar atrophy | Cerebellar atrophy |
| EMG | NA | NA |
| **Clinical Course** | | |
| Static/ Progressive | Non progressive | Non progressive |
|  |  |  |
| **Miscellaneous** | Nystagmus | Nystagmus |
| **Genetic Testing** | | |
| c.3019G>A; p.(Ala1007Thr) Homozygous | | |

**Family 3:** c.2768A>C; p.His923Pro

The first child of non-consanguineous healthy Spanish parents presented with severe hypotonia requiring resuscitation at birth. Neurological exam in the neonatal ICU revealed a weak cry with no visual tracking, severe hypotonia, absence of anti-gravity movements, and areflexia. He showed to have electroclinical seizures needing antiepileptic medications. Extensive biochemical and metabolic testing was negative. EMG/NCV showed normal latency, amplitude, and velocity of the sensory nerve. The motor nerve conductions study revealed a significant reduction of amplitude with normal latency and velocities in the nerves of the upper limbs. In the lower limbs, the motor nerves were unexcitable. All muscles explored showed a severe neurogenic pattern with abundant spontaneous activity at rest with fibrillations and positive waves.

While clinical and electromyographic data guided the diagnosis of Spinal Muscular Atrophy, the genetic study of *SMN1* was normal. At age six-months, brain MRI showed vermis predominant cerebellar atrophy which was also not in his previous MRI. After extensive panel and single gene testing, WES revealed a homozygous *GEMIN5* variant, p.(His923Pro).

On subsequent follow up, he continued to be significantly hypotonic with severe motor delay and areflexia. He also presented with microcephaly, epilepsy, and cognitive delay. At 30 months of life, he presented with a febrile illness and deteriorated rapidly with cardiorespiratory arrest, from which he could not be resuscitated.

**Table. Clinical information and supplementary investigations**

|  | Patient 4 |
| --- | --- |
| Gender | M |
| Age of symptom onset | Neonatal |
| Age at last observation | 2 years |
| **Development** | |
|  | Delay, never achieved head control, sitting or standing position, severe global development delay |
| Delayed? Y/N | Y |
| Regression Y/N | N |
| Motor delay | Y |
| Speech delay | Y |
| Cognitive delay | Y |
| **Neurological findings** | |
| Ataxia | N |
| Hypertonia | N |
| Central Hypotonia | Y (Central and peripherical hypotonia) |
| Deep tendon reflexes | N |
| Gait | Never achieved |
| **Neurological evaluation** | |
| MRI | Cerebellum atrophy |
| EMG | Electrophysiological testing: sensory nerve conductions showed normal latency, amplitude and velocity. The motor nerve revealed a significant reduction amplitude CMAPs and normal latency and velocities in upper limbs, in lower limbs the motor nerves were unexcitable. The Electromyography showed in all muscles explored a severe neurogenic pattern with abundant spontaneous activity at rest (fibrillations and positive waves were present). |
| **Clinical Course** | |
| Static/ Progressive | Progressive |
|  |  |
| **Miscellaneous** | Epilepsy |
| **Genetic Testing** | c.2768A>C; p.(His923Pro) Homozygous |

**Family 4:** c.2738A>G; p.His913Arg

The first- child of consanguineous parents of Turkish origin was born after an uneventful pregnancy. He was hospitalized on the second day of life due to hypotonia and respiratory distress. Clinical exam was notable for absent reflexes and mild contractures in the elbows and hips. Marked cerebellar atrophy was seen on brain MRI. EMG suggested pathological spontaneous activity and slight neurogenic changes of action potentials. Motor nerve conduction was markedly reduced. Sensory nerve potentials could not be elicited. The infant died at three months of age from respiratory insufficiency. Muscle biopsy was notable for large group atrophy and sural nerve displayed loss of large myelinated axons, with no evidence of demyelinating neuropathy.

In a subsequent pregnancy, a second affected boy with identical clinical picture died at two-weeks of age from respiratory insufficiency. Postmortem examination was declined. WES revealed a novel homozygous *GEMIN5* variants, p.(His913Arg) in both children.

**Table. Clinical information and supplementary investigations**

|  | Patient 5 | Patient 6 |
| --- | --- | --- |
| Gender | Male | Male |
| Age of symptom onset | Birth | Birth |
| Age at last observation | 3 months | 3 months |
| **Development** | | |
| Delayed? Y/N | Yes | Yes |
| Regression Y/N | No | No |
| Motor delay | Yes | Yes |
| Speech delay | N/A | N/A |
| Cognitive delay | N/A | N/A |
| **Neurological findings** | | |
| Ataxia | N/A | N/A |
| Hypertonia | No | No |
| Central Hypotonia | Yes | Yes |
| Deep tendon reflexes | Absent | absent |
| Gait | N/A | N/A |
| **Neurological evaluation** | | |
| MRI | Cerebellar atrophy | Cerebellar atrophy |
| EMG | pathological spontaneous activity and slight neurogenic changes of action potentials. Motor nerve conduction was markedly reduced. Sensory nerve potentials could not be elicited |  |
|  |  |  |
| **Clinical Course** | Passed away at 3 months of age | Passed away at 3 months of age |
| Static/ Progressive | N/A | N/A |
| **Miscellaneous** |  |  |
| **Genetic Testing** | c.2738A>G; p.(His913Arg) Homozygous | |

**Family 5:** c.2112 C>G; p.Asp704Glu

An 11-month-old male born to non-consanguineous parents of South Asian origin was evaluated for motor and mild cognitive delay. He presented with hypotonic and ataxic on the clinical exam. Brain MRI revealed severe cerebellar atrophy. Nerve conduction studies were normal, but needle electromyography (EMG) recordings were abnormal in the distal lower limbs with increased duration and amplitude of motor unit potentials (MUAPs) with preserved recruitment. These results were reminiscent of Spinal Muscular Atrophy, Lower Extremity Dominant (SMA-LED). Novel homozygous *GEMIN5* variants, p.(Asp704Glu), was detected on WES. His clinical and radiological exams have remained stable on follow up.

**Table. Clinical information and supplementary investigations**

|  | Patient 7 |
| --- | --- |
| Gender | Male |
| Age of symptom onset | Infancy |
| **Development** | |
| Delayed? Y/N | Y |
| Regression Y/N | N |
| Motor delay | Y |
| Speech delay | Y |
| Cognitive delay | Y |
|  |  |
| **Neurological findings** | |
| Ataxia | Y |
| Hypertonia | N |
| Central Hypotonia | Y |
| Deep tendon reflexes | ? |
| Gait | Ataxia |
| **Neurological evaluation** | |
| MRI | Cerebellar atrophy |
| EMG | Lower extremities with increased duration and amplitude of motor unit potentials (MUAPs) with preserved recruitment |
| **Clinical Course** |  |
| Static/ Progressive | Stable |
|  | |
| **Miscellaneous** |  |
| **Genetic Testing** | |
| c.2112 C>G; p.(Asp704Glu) Homozygous | |

**Family 6:** c.2049C>T; p.Gly683Asp

Two male siblings born to healthy consanguineous Egyptian parents with 2 previous normal male children were evaluated for developmental delay. The 9-year-old older sibling had a history of severe motor delay with moderate cognitive impairment. While he was able to sit up with support and use his hands, he was extremely unsteady. Neurological exam was significant for spasticity in the lower extremities along with brisk reflexes, and head nodding with intentional tremor. MRI brain showed remarkable cerebellar atrophy and EMG/NCV identified a neurogenic pattern, with reduction of amplitude of motor nerves rather than sensory, but mild delayed latencies in the upper limbs. The 7-year-old younger sibling could walk with support, though unsteady, and presented with head nodding and intentional tremor. His brain MRI showed cerebellar atrophy and EMG. His nerve conduction identified a motor neurogenic pattern in the lower limbs. A novel homozygous *GEMIN5* variant, c.2049C>T; p(Gly683Asp), was detected on WES.

**Table. Clinical information and supplementary investigations**

|  | Patient 8 | Patient 9 |
| --- | --- | --- |
| Gender | Male | Male |
| Age of symptom onset | <1yr | <1yr |
| Age at last observation | 9y 8m | 7y 6m |
| **Development** | | |
| Delayed? Y/N | Y | Y |
| Regression Y/N | N | N |
| Motor delay | Severe, can sit with support | severe, can walk with support |
| Speech delay | severe | severe, |
| Cognitive delay | moderate | mild |
| **Neurological findings** | | |
| Ataxia | Y | Y |
| Hypertonia | Y | Y |
| Central Hypotonia | Y | Y |
| Deep tendon reflexes | Brisk | Brisk |
| Gait | Non - ambulatory | Unsteady – upto 2 steps |
|  |  |  |
| **Neurological evaluation** | | |
| MRI | Cerebellar atrophy | Cerebellar Atrophy |
| EMG and NC | Neurogenic pattern, with reduction of amplitude of motor nerves rather than sensory, but mild delayed latencies in upper limbs | Neurogenic pattern, with reduction of amplitude of motor nerves rather than sensory, but normal latencies in upper limbs |
| **Clinical Course** | | |
| Static/ Progressive | Non progressive | Non progressive |
|  |  |  |
| **Miscellaneous** | Nystagmus | Nystagmus |
| **Genetic Testing** | | |
| GEMIN5: c.2049C>T p.(Gly683Asp) | | |

**Family 7:** c.2995T>C; p. Ser1000Pro

Two female siblings born full term to consanguineous Egyptian healthy parents, with no family history of neurological diseases were evaluated for developmental concerns. The elder sister, currently 9 years old, had a history of delays since infancy. Her exam revealed head nodding and nystagmus as an infant along with severe motor delays. At 9 years of age, she knew her body parts, obeyed simple commands, spoke a few words, but was non ambulatory with severe unsteadiness and tremors. Neurological exam was notable for intention tremor and dysmetria on her upper extremities with severe ataxia, hypertonia of her lower extremities with brisk reflexes, and positive Babinski sign. Sensory examination was normal. MRI brain revealed progressive cerebellar atrophy on serial imaging. EMG/NCV were normal. Her younger sister had a similar clinical course, showed to have head nodding and tremors in infancy along with motor delay. At age 4, she could take a few steps but was significantly unsteady. Neurological exam was notable for intention tremor, central hypotonia with brisk reflexes and positive Babinski sign. Neuroimaging showed mild cerebellar atrophy. EEG and EMG/ NVC were normal. Novel homozygous *GEMIN5* variants c.2995T>C; p.(Ser1000Pro) was detected on exome sequencing.

**Table. Clinical information and supplementary investigations**

|  | Patient 10 | Patient 11 |
| --- | --- | --- |
| Gender | Female | Female |
| Age of symptom onset | <1 | <1 |
| Age at last observation | 9y | 4 4/12y |
| **Development** | | |
| Delayed? Y/N | Y | Y |
| Regression Y/N | N | N |
| Motor delay | Severe | severe |
| Speech delay | + few words | + words |
| Cognitive delay | Y | Y |
| **Neurological findings** | | |
| Ataxia | + | + |
| Hypertonia | + | + |
| Central Hypotonia | - | - |
| Deep tendon reflexes | Brisk | brisk |
| Gait | Non ambulatory | non ambulatory |
| **Neurological evaluation** | | |
| MRI | Cerebellar atrophy | Mild cerebellar atrophy |
| EMG | Normal | Normal |
| **Clinical Course** | | |
| Static/ Progressive | Static | Static |
| **Miscellaneous** |  | strabismus |
| **Genetic Testing** | | |
| GEMIN5: c.2995T>C; p.(Ser1000Pro) Homozygous | | |

**Family 8:** c.2995T>C; p. Ser1000Pro

Two male Egyptian decent siblings were born to healthy consanguineous Egyptian parents, with no family history of neurological issues. The older brother is currently 10 years old. He showed nystagmus at 2 months of age and subsequently presented with severe motor delays. He was able to support his head and sit with support at 2 years of age. Quadriparesis was observed and cognitive functions showed ability to vocalize, say letters, recognize the surrounding, but were delayed for age. The course was static. His clinical exam showed a generalized wasting with nystagmus, intention tremor with foot, and hand drop. Reflexes were present but less distally. MRI Brain revealed cerebellar atrophy and EMG/ NVC showed possible affection of anterior horn cells.

The younger brother at age 2 was more severely affected. He was unable to sit up with support and experienced head lag at 2 years of age. His exam showed a severe hypotonia with preserved reflexes. MRI Brain at age 2 showed mild cerebellar atrophy and EMG/ NCV showed similar suggestion of anterior horn cell affliction.

**Table. Clinical information and supplementary investigations**

|  | Patient 12 | Patient 13 |
| --- | --- | --- |
| Gender | Male | Male |
| Age of symptom onset | 2months, nystagmus | 2months, nystagmus |
| Age at last observation | 10y | 2y |
| **Development** | | |
| Delayed? Y/N | Y | Y |
| Regression Y/N | N | N |
| Motor delay | Severe, couldn’t sit | Severe, couldn’t sit |
| Speech delay | words | No speech |
| Cognitive delay | Y | Y |
| **Neurological findings** | | |
| Ataxia | + intention tremors, nystagmus | Couldn’t assessed (quadriplegia), nystagmus |
| Hypertonia | - |  |
| Central Hypotonia | + | + |
| Deep tendon reflexes | present | present |
| Gait | Unable to walk | Unable to walk |
| **Neurological evaluation** | | |
| MRI | Cerebellar atrophy | Cerebellar atrophy |
| EMG | Neuropathic, fibrillation potentials, denervation, and increased amplitude suggestive of anterior horn cells affection | Neuropathic, fibrillation potentials, denervation, and increased amplitude suggestive of anterior horn cells affection |
| **Clinical Course** | | |
| Static/ Progressive | static | static |
| **Miscellaneous** | Wasting, claw hands, acquired mild arthrogryposis | Wasting, claw hands, plantar flexion of feet, VSD |
| **Genetic testing** | | |
| GEMIN5: c.2995T>C; p.(Ser1000Pro) Homozygous | | |

**Family 9: (**c.1831G>A; p.His1364Pro, c.4091A>C; p.Val611Met

**Val611Met/His1364Pro**

**Val611Met/WT**

**His1364Pro/WT**

Patient 14


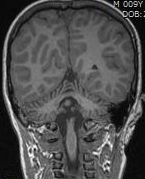

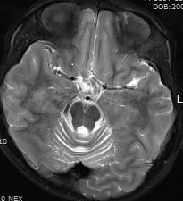

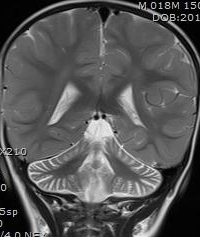

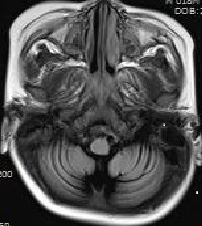


A 14-year-old male and his 6-year-old brother, born to non-consanguineous parents of East Asian origin, were evaluated for developmental delay. Neurological exam showed hypotonia, dysmetria, truncal ataxia, and cognitive impairment. Brain MRI showed cerebellar atrophy. WES revealed compound heterozygous variants in *GEMIN5,* c.1831G>A; p. (His1364Pro), and, c.4091A>C; p.(Val611Met).

**Table. Clinical information and supplementary investigations**

|  | Patient 14 | Patient 15 |
| --- | --- | --- |
| Gender | M | M |
| Age of symptom onset | <1 yr | 10 months |
| **Development** | | |
| Delayed? Y/N | Y | Y |
| Regression Y/N | N | N |
| Motor delay | Y | Y |
| Speech delay | Y | Y |
| Cognitive delay | Y | Y |
| **Neurological findings** | | |
| Ataxia | Y | N/A |
| Hypertonia | N | N |
| Central Hypotonia | Y | Y |
| Deep tendon reflexes |  |  |
| Gait | ataxia | N/A |
| **Neurological evaluation** | | |
| MRI | Cerebellar atrophy | Cerebellar atrophy |
| EMG |  |  |
| **Clinical Course** |  |  |
| Static/ Progressive |  |  |
|  |  |  |
| **Miscellaneous** |  |  |
| **Genetic Testing** | | |
| c.1831G>A; p.(His1364Pro) Heterozygous  c.4091A>C; p.(Val611Met) Heterozygous | |  |

**Family 10:** c.2510-16_2523inv CTTGATTAAGGTTTCTGAAATTTAATAAAG (splicing acceptor), c.3857A>G; p. Tyr1286Cys

**2510-2A>T/ Tyr1286Cys**

**2510-2A>T/WT**

**Tyr1286Cys/WT**

A four-year-old female, born to non-consanguineous parents of East Asian origin, was evaluated for a history of developmental delay present since she was around six-months of age. At four-years old, she became ambulatory but unsteady. She followed simple commands and had a vocabulary of 10-20 words. Neurological exam showed central hypotonia, tremor, and ataxia with brisk reflexes. Brain MRI revealed severe cerebellar atrophy. WES revealed compound heterozygous variants in *GEMIN5,* c.2510-16_2523invCTTGATTAA GGTTTCTGAAATTTAATAAAG splicing acceptor, and, c.3857A>G; p.(Tyr1286Cys).

**Table. Clinical information and supplementary investigations**

|  | Patient 16 |
| --- | --- |
| Gender | F |
| Age of symptom onset | 6 months |
| Age at last observation | 4 years |
| **Development** | |
| Delayed? Y/N | Y |
| Regression Y/N | N |
| Motor delay | Y |
| Speech delay | Y |
| Cognitive delay | Y |
| **Neurological findings** | |
| Ataxia | Y |
| Hypertonia | N |
| Central Hypotonia | Y |
| Deep tendon reflexes | Brisk |
| Gait | Ataxia |
| **Neurological evaluation** | |
| MRI | Cerebellar Atrophy |
| EMG |  |
| **Clinical Course** |  |
| Static/ Progressive |  |
|  | |
| **Miscellaneous** |  |
| **Genetic Testing** | |
| c.2510-16_2523inv CTTGATTAAGGTTTCTGAAATTTAATAAAG, splicing acceptor Heterozygous  c.3857A>G; p.(Tyr1286Cys) Heterozygous | |

**Family 11:** c.2962A.T; p.lle988Phe, c.4100T.C; p.Leu1367Pro

A 15-year-old boy was followed for history of developmental delays, generalized weakness, muscular atrophy, contractures, and epilepsy. Brain MRI showed cerebellar atrophy along with volume loss in the pons/ brainstem. His EMG findings were consistent with those of a long-standing disorder of motor neurons. WES revealed compound heterozygous *GEMIN5* variants, p.(l988F) (paternal), and, p.(Leu1367Pro) (maternal).

**Table. Clinical information and supplementary investigations**

**Leu1367Pro/WT**

**lle988Phe/WT**

**lle988Phe/**

**Leu1367Pro**

|  | Patient 17 |
| --- | --- |
| Gender | Male |
| **Development** | |
| Delayed? Y/N | Y |
| Regression Y/N | N |
| Motor delay | Y |
| Speech delay | Y |
| Cognitive delay | Y |
| **Neurological findings** | |
| Ataxia | Y |
| Hypertonia |  |
| Central Hypotonia | Y |
| Deep tendon reflexes |  |
| Gait |  |
| **Neurological evaluation** | |
| MRI | Pontocerebellar Atrophy |
| EMG | Consistent with long-standing disorder of motor neurons |
| **Clinical Course** |  |
| Static/ Progressive | Progressive |
| **Miscellaneous** |  |
| **Genetic Testing** | |
| c.2962A.T; p.(lle988Phe) Heterozygous  c.4100T.C, p.(Leu1367Pro) Heterozygous | |

**Family 12:** c.4100T>C; p.Leu1367Pro, c.3057C>A; p.Asp1019Glu

**Leu1367Pro/WT**

**Asp1019Glu/WT**

**Leu1367Pro/Asp1019Glu**

An eight-year-old female born to non-consanguineous parents of Caucasian origin was followed for a history of developmental delays with exam notable for diffuse hypotonia, motor delay, mild cognitive delay, brisk reflexes, ataxia, dysarthria, slowed visual pursuit, and mild oculomotor apraxia. EMG was normal. Brain MRI showed moderate to severe cerebellar volume loss. WES revealed compound heterozygous *GEMIN5* variants, c.4100T>C p.(Leu1367Pro); and c.3057C>A p.(Asp1019Glu). She developed seizures consistent with benign epilepsy of childhood with centrotemporal spikes (BECTS) at 6 years of age. Her younger brother, now six years of age, showed motor delay, tremor, ataxia, hyperreflexia and mild dysarthria, and was also found to be compound heterozygous for these variants. His MRI later showed mild to moderate cerebellar volume loss.

**Table. Clinical information and supplementary investigations**

|  | Patient 18 | Patient 19 |
| --- | --- | --- |
| Gender | Female | Male |
| Age of symptom onset | 7 mo (motor development slowed); 12 mo tremor, imbalance and frequent falls | 2 yrs (motor development slowed); 3 yrs tremor and imbalance |
| Current development | Ambulatory mostly with walker, short distances with braces only; fine motor delay, some cognitive delay. | Ambulatory but mild gross motor delay. Normal cognition |
| **Development** | | |
| Delayed? Y/N | Y | Y |
| Regression Y/N | N | N |
| Motor delay | Delayed | Delayed |
| Speech delay | Y | Y |
| Cognitive delay | Y | N |
| **Neurological findings** | | |
| Ataxia | Y | mild |
| Hypertonia | Y | mild |
| Central Hypotonia | Y | Mild |
| Gait | Ataxic | Mild Ataxic |
| Tremor/ abnormal movements | Y | Y |
| **Neurological evaluation** | | |
| MRI | Cerebellar volume loss, moderate to severe | Cerebellar volume loss, mild to moderate |
| EMG | Normal | Not done |
| **Clinical Course** |  |  |
| Static/ Progressive | Static | Static |
|  |  |  |
| **Miscellaneous** | Dysarthria, slow visual pursuit, mild oculomotor apraxia, hyperreflexia, extensor plantar response, seizures | Mild dysarthria, hyperreflexia, extensor plantar response |
| **Genetic testing** | | |
| c.4100T>C; p.(Leu1367Pro) Heterozygous  c.3057C>A; p.(Asp1019Glu) Heterozygous | | |

**Family 13:** c.3844 T>C; p.Tyr1282His, c.217 T>C; p.Ser73Pro

A seven-year-old Caucasian male presented in infancy with hypotonia, bilateral cataracts, and global delays. Neurological exam showed central hypotonia, ataxia, and spasticity with bilateral ankle with upgoing toes. MRI showed a progressive cerebellar atrophy with mild cerebellar cortex T2 hyperintensity. EMG was not completed. He presented with seizures and an EEG showed bilateral Centro-temporal spikes. Ataxia expanded panel analysis revealed two heterozygous *GEMIN5* variants, c.3844 T>C; p.(Tyr1282H), and, c.217 T>C; p.(Ser73Pro).

**Ser73Pro/WT**

**Tyr1282His/WT**

**Tyr1282His/**

**Ser73Pro**

**Table. Clinical information and supplementary investigations**

|  | Patient 20 |
| --- | --- |
| Gender | Male |
| Age of symptom onset | Birth |
| Age at last observation | 7 years |
| **Development** | |
| Delayed? Y/N | Y |
| Regression Y/N | N |
| Motor delay | Y |
| Speech delay | Y |
| Cognitive delay | Y |
| **Neurological findings** | |
| Ataxia | Y |
| Hypertonia | Y |
| Central Hypotonia | Y |
| Deep tendon reflexes | Increased with clonus at both ankles, upgoing toes bilaterally |
| Gait | Ataxic with wide base and spastic, requiring use of walker |
|  | Distal weakness in the legs>arms |
| **Neurological evaluation** | |
| MRI | 2018:   \|  \| \| --- \| \| Significant progression in cerebellar atrophy, now moderate to severe.  Mild cerebellar cortex T2 hyperintensity. \| \|  \| \| No new signal abnormality or supratentorial volume loss.  2014:   \|  \| \| --- \| \| Mild-moderate cerebellar atrophy. \| \|  \| \| Changes related to bilateral cataract repair. \| \| |
| EMG | None |
| EEG | EEG SUMMARY:  1. Mild diffuse background slowing  2. Spikes, bilateral centro-temporal, frequent |
| **Clinical Course** | |
| Static/ Progressive | Progressive, with development of seizures |
| **Miscellaneous** | |
|  | Has cataracts and seizures |
|  |  |
| **Genetic testing** | GEMIN5  c.3844 T>C; p.(Tyr1282His) Heterozygous  c.217 T>C; p.(Ser73Pro) Heterozygous |

**Family 14** (c.3353 T>C; p.Leu1119Ser, c.1599C>A; p.Tyr534Ter

A 31-year-old male of Guyanese descent followed since 1.5 years of age for gross motor delays. He presented with a severe axial hypotonia and brisk reflexes. He was diagnosed with cataracts at one-year old and they were extracted at six-years old. He is currently wheelchair bound due to severe ataxia and has an exam notable for spasticity in all four extremities with mild impairment in cognition. Recent MRI Brain showed vermian more than hemispheric cerebellar atrophy, which stable compared to his previous imaging in childhood. WES revealed compound heterozygote variants in *GEMIN5*.

**Table. Clinical information and supplementary investigations**

|  | Patient 21 |
| --- | --- |
| Gender | Male |
| Age of symptom onset | Presented at 1.5 yr due to prominent gross motor delay |
| Age at last observation | 31 yrs |
| **Development** | |
| Delayed? Y/N | Y |
| Regression Y/N | N |
| Motor delay | Moderate |
| Speech delay | Mild |
| Cognitive delay | Mild |
| **Neurological findings** | |
| Ataxia | Axial and appendicular, severe |
| Hypertonia | Y |
| Central Hypotonia | Y |
| Deep tendon reflexes | 3+ throughout |
| Gait | Ataxic, largely wheelchair bound |
| **Neurological evaluation** | |
| MRI | Cerebellar atrophy |
| EMG | None |
| **Clinical Course** |  |
| Static/ Progressive | Static |
| **Miscellaneous** | Infantile cataracts, distal weakness prominent in UL |
| **Genetic testing** | |
| c.3353 T>C; p.(Leu1119Ser) Heterozygous  c.1599C>A; p.(Tyr534Ter) Heterozygous | |

**Family 15** c.2962A>T; p.lle988Phe, c.1856delA; p.Glu619GlyfsTer8

**Ile988Phe/WT**

**Glu619GlyfsTer/WT**

**Glu619GlyfsTer/**

**Ile988Phe**

A ten-month-old Caucasian male was evaluated for developmental delay. Exam was notable for hypotonia, poor visual tracking, and brisk reflexes. No regression was observed to date. Brain MRI revealed cerebellar atrophy around 25 months. Electromyogram (EMG) and nerve conduction studies (NCS) showed a myopathic process with large motor units from the tibialis anterior. WES revealed compound heterozygous *GEMIN5* variants, c.2962A>T; p.(lle988Phe) (maternal), and, c.1856delA; p.Glu619GlyfsTer8 (Paternal).

**Table. Clinical information and supplementary investigations**

|  | Patient 22 |
| --- | --- |
| Gender | Male |
| Age of symptom onset | <1 y |
| **Development** | |
| Delayed? Y/N | Yes |
| Regression Y/N | No |
| Motor delay | Yes |
| Speech delay |  |
| Cognitive delay |  |
| **Neurological findings** | |
| Ataxia | Not walking yet |
| Hypertonia | No |
| Central Hypotonia | Yes |
| Deep tendon reflexes | Brisk |
| Gait | Not walking yet |
| **Neurological evaluation** | |
| MRI | Cerebellar atrophy |
| EMG | Myopathic process with large motor units from tibialis anterior |
| **Clinical Course** |  |
| Static/ Progressive |  |
| **Miscellaneous** |  |
| **Genetic testing** | |
| c.2962A.T p. lle988Phe Heterozygous  c.1856delA p.Glu619GlyfsTer8 Heterozygous | |

**Family 16** c.314A>G; p.His105Arg, c.2768A>C; p.His923Pro

This seven-year-old female was first seen at one-year of age for concern about motor delay. She was born full term by C section for maternal pre-eclampsia. She showed hypotonia and tremulousness on exam. MRI revealed pronounced cerebellar atrophy. EMG was not completed. WES revealed a novel compound heterozygote variant of the *GEMIN5* gene*,* c.314A>G; p.(His105Arg), in the patient.

**Table. Clinical information and supplementary investigations**

**His105Arg/WT**

**His923Pro/WT**

**His105Arg/His923Pro**

|  | Patient 23 |
| --- | --- |
| Gender | Female |
| Age of symptom onset | 1 year |
| Age at last observation | 7 year |
| **Development** | |
| Delayed? Y/N | Yes |
| Regression Y/N | No |
| Motor delay | Yes |
| Speech delay | Yes |
| Cognitive delay | Yes |
| **Neurological findings** | |
| Ataxia | Yes |
| Hypertonia | No |
| Central Hypotonia | Yes |
| Deep tendon reflexes |  |
| Gait | ataxic |
| **Neurological evaluation** | |
| MRI | Cerebellar atrophy |
| EMG | Not done |
|  |  |
| **Clinical Course** |  |
| Static/ Progressive |  |
| **Miscellaneous** |  |
| **Genetic testing** | |
| c.314A>G; p.(His105Arg) Heterozygous  c.2768A>C; p.(His923Pro) Heterozygous | |

**Family 17** c.2962A>T; p.Ile988Phe, c.3930_3933delCTCT; p. Ser1311LeufsTer7

**Ser1311LeufsTer7/WT**

**Ile988Phe/WT**

**Ile988Phe/**

**Ser1311LeufsTer7**

Two brothers, now 29 and 27 years old, were followed from infancy for hypotonia and developmental delay. The 29-year-old patient was followed for hypotonia at four months and ataxia since one year of age. He started having seizures around five years of age. Clinical examination showed weakness, appendicular hypertonia, and myoclonic action tremor. Brain MRI showed progressive cerebellar atrophy. EMG was not completed. EEG revealed slow background with no epileptiform discharges.

The brother, now 27-years old, was hypotonic at birth. He was walking with a walker until six years of age and has been non ambulatory since he turned 14. Clinical exam showed appendicular hypertonia, weakness, brisk reflexes in the upper extremities, and patella with absent reflexes in the ankles. Brain MRI showed progressive cerebellar atrophy. EMG/ NCV showed chronic loss of anterior horn cells but no abnormal spontaneous activity to support ongoing denervation. Repeat EMG/NCV ten years later showed moderate chronic and ongoing motor neuropathy or motor neuronopathy. EEG showed diffuse slowing with no epileptiform discharges. WES revealed compound heterozygous *GEMIN5* variants, c.2962A>T; p.(Ile988Phe) (Paternal), and, c.3930_3933delCTCT; p. (Ser1311LeufsTer7) (Maternal).

. **Table. Clinical information and supplementary investigations**

|  | Patient 24 | Patient 25 |
| --- | --- | --- |
| Gender | Male | Male |
| Age of symptom onset | 4 months of age with hypotonia  Ataxia observed since at least 1 year of age, Seizures at 5-6 years of age | Birth with hypotonia |
| Age at last observation | 29 years | 27 years |
| **Development** | | |
| Delayed? Y/N | Y | Y |
| Regression Y/N | N | N |
| Motor delay | Y | Y |
| Speech delay | Y | Y |
| Cognitive delay | Y | Y |
| **Neurological findings** | | |
| Ataxia | Y | Y |
| Hypertonia | Y | Y |
| Central Hypotonia | N | N |
| Deep tendon reflexes | 3+ in the biceps, triceps, brachioradialis, patella and 2+ at the achilles. | 3+ in the biceps, triceps, brachioradialis, patella and absent at the achilles. |
| Gait | Nonambulatory since early childhood | Walked with walker until 6 years of age, then nonambulatory since 14 years of age. |
|  | Myoclonic action tremor  Significant muscle atrophy throughout. Antigravity strength in the arms and legs | Myoclonic tremor in the arms. Slow movements in the hands. Large amplitude saccades were hypometric. Breakdown of smooth pursuit. |
| **Neurological evaluation** | | |
| MRI | MRI brain 2016:  Progression of cerebellar volume loss since 2007, now moderate to severe, with slight T2 hyperintensity along the  cerebellar cortex.  New mild diffuse cerebral volume loss.  MRI brain 2007:  Impression:  No supratentorial abnormality seen.  Atrophy of the cerebellar hemispheres/vermis | MRI brain: 2016:  IMPRESSION:  Progressive cerebellar volume loss, now moderate-severe.  MRI brain 2009:  Impression: Microcephaly with diffuse cerebral volume loss and marked cerebellar volume loss. |
| EMG | None | - EMG (4/9/03, Shriner’s Hospital) - Abnormal study. These findings support a possible chronic involvement of muscle as part of this multisystemic process. Chronic loss of anterior horn cells could also result in a similar pattern, but there is no abnormal spontaneous activity to support active ongoing denervation, if this were the case. Recommend muscle biopsy right gastrocnemius. - EMG/NCV, 9/29/13 (Children’s Hospital Colorado): Abnormal study. The electrophysiologic findings are most suggestive of a moderate chronic and ongoing motor neuropathy or motor neuronopathy.  An incidental note is made of a median neuropathy at the right wrist (as in carpal tunnel syndrome). |
| EEG | EEG 2014: IMPRESSION: This is an abnormal EEG during awake and sleep stages due to  background mildly diffused slowing. This is consistent with mild   cerebral dysfunction. No clinical or electrographic seizures were  recorded. | EEG: 2014 IMPRESSION: This is an abnormal EEG during awake and sleep stages due to  diffused slowing. This is consistent with mild cerebral  dysfunction. No clinical or electrographic seizures were  recorded. |
| **Clinical Course** | | |
| Static/ Progressive | Progressive with worsening ataxia, weakness, and spasticity | Progressive with loss of ambulation, increased spasticity, progressive scoliosis, night blindness |
| **Miscellaneous** | | |
|  | Has stable generalized seizures controlled with AEDs | Has stable generalized seizures controlled with AEDS |
|  |  |  |
| **Genetic testing** | GEMIN5  c.2962 A>T; (p.Ile988Phe) (father)  c.3930_3933delCTCT; (p.Ser1311Leu fsX7) (mother) | |

**Family 18** c.485A>G; p.His162Arg, c.4100T>C; p.Leu1367Pro

**Leu1367Pro/WT**

**His162Arg/WT**


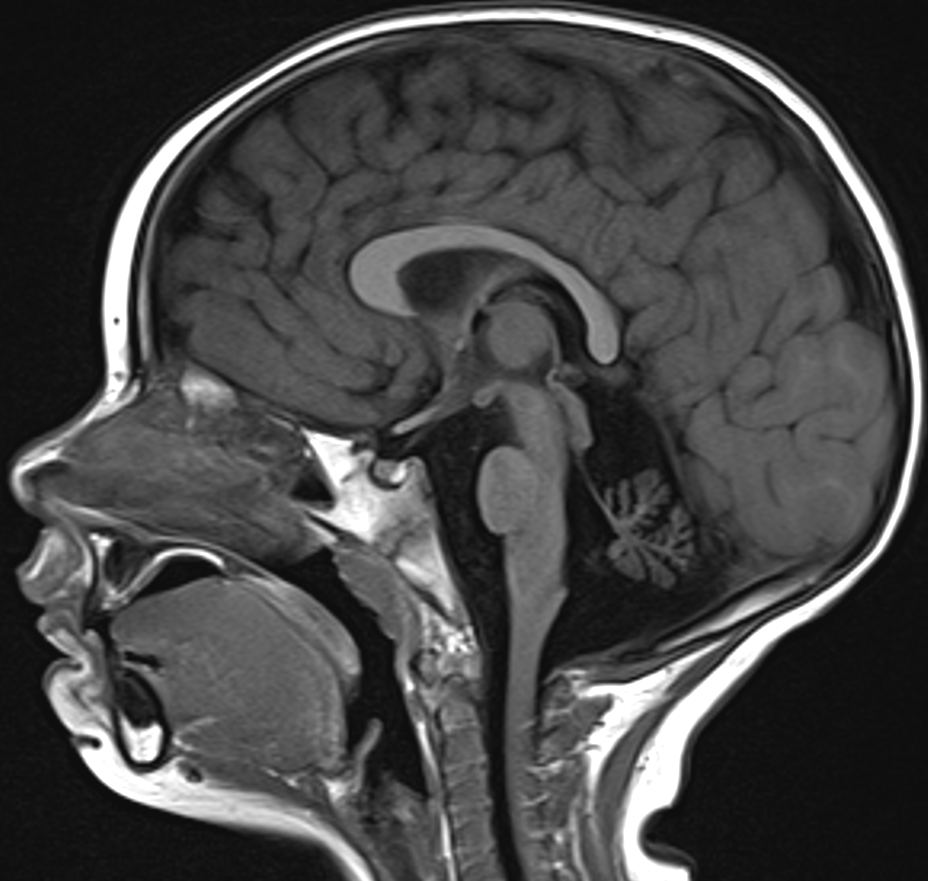

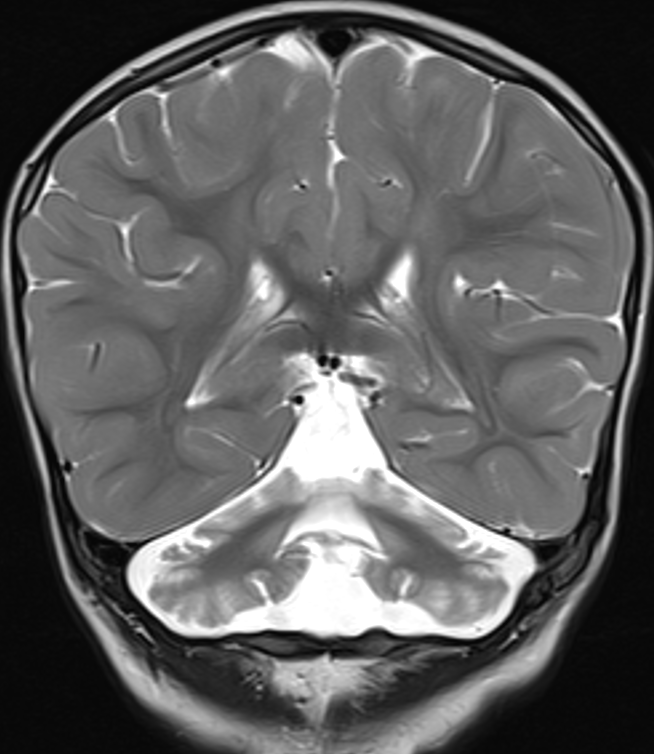


**Leu1367Pro/**

**His162Arg**

An 18-month-old female was evaluated for developmental delay, hypotonia, seizures, and suspicion for motor neuron disease. Exam was notable for severe central hypotonia with absent reflexes. EMG was consistent with severe neuropathic disorder. Initial MRI was reported to be WNL. WES revealed compound heterozygous variants in the *GEMIN5* gene, c.485A>G; p.(His162Arg), and, c.4100T>C; p.(Leu1367Pro). On subsequent follow up, she presented with slow motor functional gains and had well controlled seizures. A repeat Brain MRI showed cerebellar vermian and hemispheric atrophy.

**Table. Clinical information and supplementary investigations**

|  | Patient 26 |
| --- | --- |
| Gender | Female |
| Age of symptom onset | birth |
| Age at last observation | 18 months |
| **Development** | |
| Delayed? Y/N | Y |
| Regression Y/N | N |
| Motor delay | Y |
| Speech delay | N |
| Cognitive delay | N |
| **Neurological findings** | |
| Ataxia | N |
| Hypertonia | N |
| Central Hypotonia | Y (severe) |
| Deep tendon reflexes | Absent |
| Gait | Non-ambulatory |
| **Neurological evaluation** | |
| MRI | Normal |
| EMG | Left median, peroneal and tibial motor responses were absent. Left sural sensory nerve action potential (SNAP) was not obtainable. Needle EMG of selected muscles of the left upper and lower extremities showed abnormal spontaneous activity with fibrillation potentials and fasciculations. During voluntary activation no motor unit action potentials were seen in the lower extremities. In the biceps recruitment was reduced with large polyphasic units.  Interpretation: This is an abnormal study. There is electrodiagnostic evidence for severe neuropathic disorder. |
| **Clinical Course** |  |
| Static/ Progressive | Static |
|  |  |
| **Miscellaneous** | mildly high narrow palate; strabismus; head drop; areflexia; generalized tonic seizures starting at 5 weeks of age- seizures were difficult to control at first, but now well controlled on Clobazam; abnormal EEG |
| **Genetic testing** | c.485A>G; p.(His162Arg) Heterozygous  c.4100T>C; p.(Leu1367Pro) Heterozygous |

**Family 19:** c.282G>A; p.Trp94Ter, c.3856T>A; p.Tyr1286Asn

**Trp94ter/WT**

**Trp94ter/**

**Tyr1286Asn**

**Tyr1286Asn/WT**

The index patient is the only daughter of non-consanguineous German parents. She presented with neonatal seizures at 3 weeks of age which were treated with Levetiracetam for 6 months, but seizures never reoccurred. The parents first noticed a development delay at 3 months. MRI Brain at 10 months of age showed cerebellar atrophy most predominantly of the vermis with concomitant FLAIR hyperintensities of the cerebellar cortex. A follow up MRI at the 2 ½ years confirmed progressive cerebellar atrophy with cerebellar cortex hyperintensities. Myelination was normal. Neurological assessment revealed severe global developmental delay. The girl cannot sit or stand but can roll over. She is verbalizing repetitive syllables but no words. She has severe truncal hypotonia without head control and peripheral hypertonia with a positive Babinski’s sign, as well as repeated opisthotonos posturing. Additionally, she has bilateral hip luxation which is treated conservatively.

WES identified biallelic variants in the *GEMIN5* gene, a paternally inherited nonsense variant, c.282G>A; p.(Trp94Ter), and a missense variant, c.3856T>A; p.(Tyr1286Asn), inherited from the mother.

**Table. Clinical information and supplementary investigations**

|  | Patient 27 |
| --- | --- |
| Gender | Female |
| Age of symptom onset | 4 months |
| Age at last observation | 31 months |
| **Development** | |
| Delayed? Y/N | Yes |
| Regression Y/N | No |
| Motor delay | Yes |
| Speech delay | Yes |
| Cognitive delay | Yes |
| **Neurological findings** | |
| Ataxia | Not walking |
| Hypertonia | Yes |
| Central Hypotonia | Yes |
| Deep tendon reflexes | Brisk |
| Gait | The girl cannot sit or stand but can roll over |
|  |  |
| **Neurological evaluation** | |
| MRI | Cerebellar atrophy |
| EMG |  |
| **Clinical Course** |  |
| Static/ Progressive | Static |
|  |  |
| **Genetic testing** | |
| c.282G>A; p.(Trp94Ter) Heterozygous,  c.3856T>A; p.(Tyr1286Asn) Heterozygous | |

**Family 20** c.1081-2A>G Splice acceptor variant, c.628G>T; p.Asp210Tyr


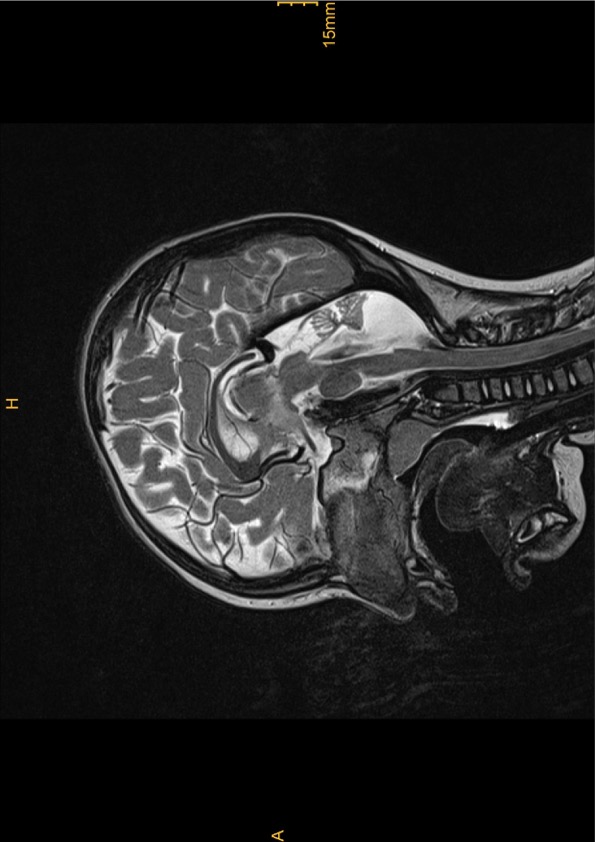


**c.1081-2A>G /**

**Asp210Tyr**

A 5-year-old boy, first son of non-consanguineous parents of Indian ancestry, was evaluated for global developmental delay. He was born by C-section at 36 weeks of gestation due to maternal eclampsia. He was noted to be “floppy” at birth but needed no pulmonary support. Subsequently, he showed a severe global developmental delay. At 19 months, exam revealed spontaneous smile and ability to roll but no head control or ability to sit. He presented with a spastic dystonic movement disorder with brisk reflexes. MRI Brain showed global atrophy, most pronounced in the cerebellar vermis. At 5 years of age he was able to control his head for some seconds and had no active speech. WES revealed novel compound heterozygote variants of the *GEMIN5* gene in the patient.

**Table. Clinical information and supplementary investigations**

|  | Patient 28 |
| --- | --- |
| Gender | Male |
| Age of symptom onset | Birth |
| Age at last observation | 5 years |
| **Development** | |
| Delayed? Y/N | Yes |
| Regression Y/N | No |
| Motor delay | No motor milestones achieved |
| Speech delay | No active speech achieved |
| Cognitive delay | Severe |
| **Neurological findings** | |
| Ataxia | Yes |
| Hypertonia | No |
| Central Hypotonia | Yes |
| Deep tendon reflexes | Hyperreflexia |
| Gait | Not possible |
| **Neurological evaluation** | |
| MRI | Cerebellar atrophy, mild cortical atrophy |
| EMG | Not performed |
| **Clinical Course** |  |
| Static/ Progressive | Static |
| **Miscellaneous** | SEP, AEP, VEP normal at age of 1 year.  Turricephalus, spastic-dystonic movement disorder, severe cachexia with BMI 11.5kg/m². |
| **Genetic testing** | |
| c.1081-2A>G, Splice acceptor variant, Heterozygous,  c.628G>T p.(Asp210Tyr) | |

**Family 21** c.4229delC; p.Ala1410GlufsTer24, c.2773C>T; p.Leu925Phe

**Ala1410GlufsTer24/WT**

**Leu925Phe/WT**

**Ala1410Glufs*24/Leu925Phe**


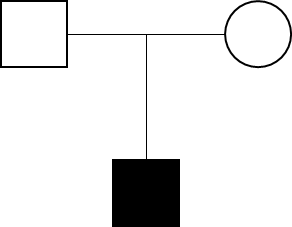


A 3-year-old boy born to non-consanguineous parents of Chinese origin presented with uncoordinated limb movements at 10 months of age. He was evaluated for developmental delay and at 3 he was unable to stand or walk without support and could talk in simple phrases. His MRI Brain was reported to as Dandy Walked malformation though on review suggested to be more of cerebellar atrophy. Exome testing revealed novel compound heterozygous variants in the GEMIN5 gene ( c.4229delC; .Ala1410GlufsTer24, c.2773C>T; p.Leu925Phe)

**Table. Clinical information and supplementary investigations**

|  | Patient 29 |
| --- | --- |
| Gender | Male |
| Age of symptom onset | Uncoordinated limbs movement was seen at 10 months. At the age of 3 years he is unable stand and walk alone without support, can talk in simple 4-5 -word phrases |
| Age at last observation | 3 years |
| **Development** | |
| Delayed? Y/N | Yes |
| Regression Y/N | No |
| Motor delay | Rising head at 3 months  Sat: 6 months  Stand with support 1year |
| Speech delay | Can speak phrases of 4-5 words but unclear |
| Cognitive delay | Delayed |
| **Neurological findings** | |
| Ataxia | Not walking |
| Hypertonia | N/A |
| Central Hypotonia | Low |
| Deep tendon reflexes | present |
| Gait | Unstable, uncoordinated |
| Nystagmus | No |
| **Neurological evaluation** | |
| MRI | Reported as Dandy walker malformation |
| EMG and NCV | Normal |
| **Clinical Course** |  |
| Static/ Progressive | Static |
| **Miscellaneous** | VEP/ ERG normal |
| **Genetic testing** | c.4229delC; p.Ala1410GlufsTer24  c.2773C>T; p.Leu925Phe |

**Family 22** c.754C>T; p.Arg252Ter, c.2872T>C; p.Tyr958His

**3 year 6 months**

**1 year 8 months**

**Arg252Ter/WT**

**Tyr958His/WT**

**Arg252Ter/Tyr958His**

**
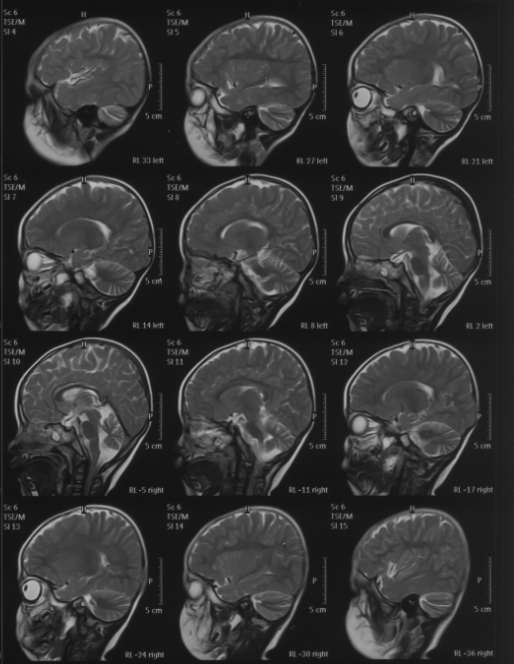

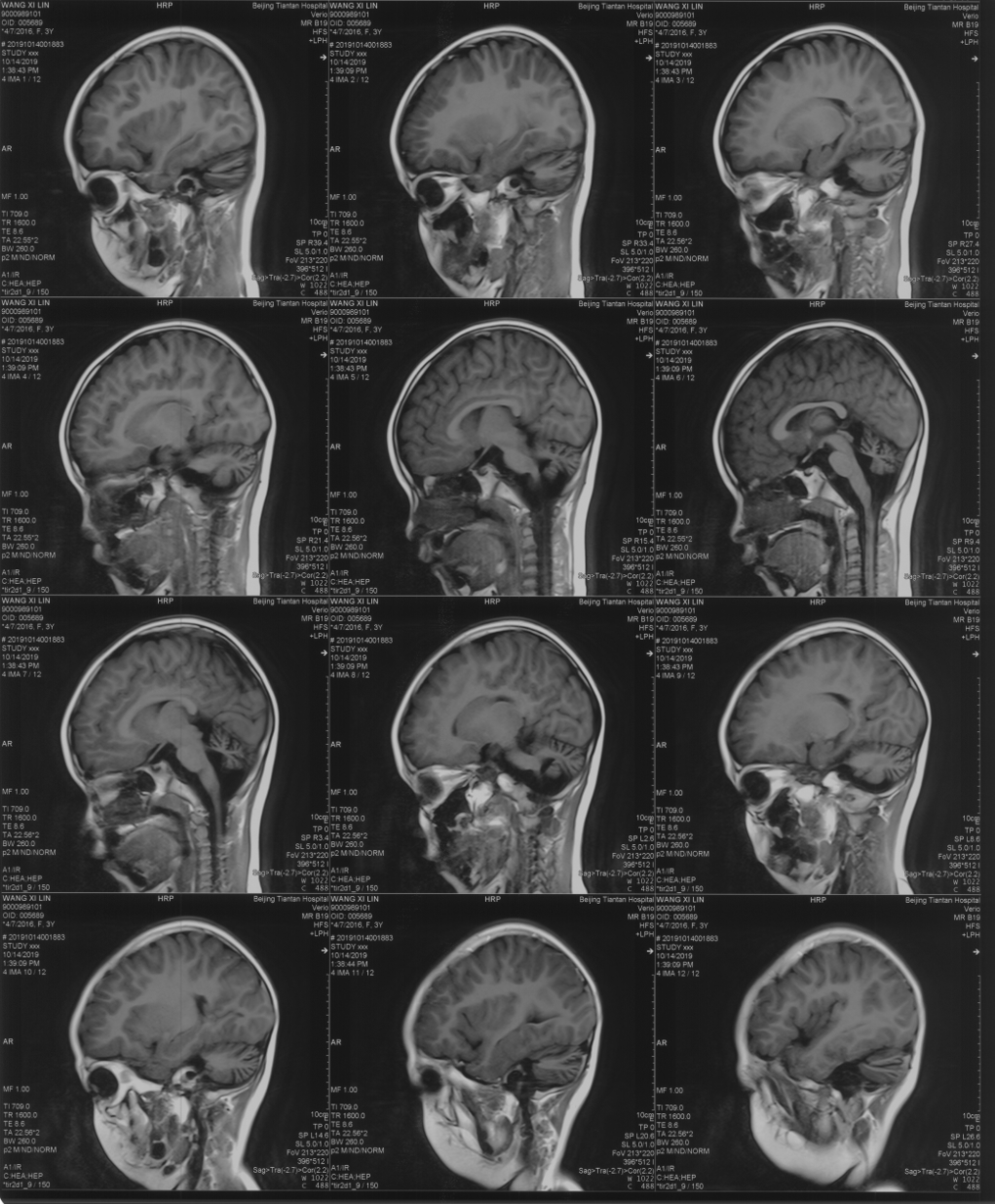
**

A 4-year-old girl was assessed for developmental delay noticed at age 1 due to an uncoordinated gait. At age 4, she was able to able to walk independently but with an ataxic gait and could talk in simple phrases. Her exam showed a positive finger nose test and an ataxia gait. MRI Brain revealed cerebellar atrophy. Exome testing revealed novel compound heterozygous variants in the GEMIN5 gene ( c.754C>T; p.Arg252Ter, c.2872T>C; p.Tyr958His)

**Table. Clinical information and supplementary investigations**

|  | Patient 30 |
| --- | --- |
| Gender | Female |
| Age of symptom onset | Uncoordinated gait was seen at 1 year 2 months. At the age of 4.5 years she could walk alone without support but is ataxic, could only talk in simple 4-5 -word phrases and identify the numbers “1-10”. |
| Age at last observation | 4 year 6 months |
| **Development** | |
| Delayed? Y/N | Yes |
| Regression Y/N | No |
| Motor delay | Yes |
| Speech delay | Can speak several phrases of 4-5 words but unclear |
| Cognitive delay | Delayed, can only express some basic needs, can identify the numbers “1-10” |
| **Neurological findings** | |
| Ataxia | Finger nose test (+) |
| Hypertonia | Normal |
| Central Hypotonia | Normal |
| Deep tendon reflexes | Present |
| Gait | Unstable, uncoordinated |
| Nystagmus | No |
| **Neurological evaluation** | |
| MRI | Cerebellar atrophy |
| EMG and NCV | Normal |
| **Clinical Course** |  |
| Static/ Progressive | Progressive |
| **Miscellaneous** | VEP/ ERG Normal |
| **Genetic testing** | c.754C>T; p.Arg252Ter  c.2872T>C; p.Tyr958His  Compound heterozygous |
