# Supplementary material for: Loss of function mutations in GEMIN5 cause a neurodevelopmental disorder
Source: Nat Commun. 2021 May 7;12:2558. doi: 10.1038/s41467-021-22627-w (PMC8105379; doi:10.1038/s41467-021-22627-w)
Supplement: Supplementary file 5 — Reporting summary [file 41467_2021_22627_MOESM5_ESM.pdf]

nature research

Corresponding author(s): Udai Bhan Pandey

Last updated by author(s): Feb 22, 2021

Reporting Summary

Nature Research wishes to improve the reproducibility of the work that we publish. This form provides structure for consistency and transparency in reporting. For further information on Nature Research policies, see our Editorial Policies and the Editorial Policy Checklist.

Statistics

For all statistical analyses, confirm that the following items are present in the figure legend, table legend, main text, or Methods section.

n/a	Confirmed	
☐	☒	The exact sample size (*n*) for each experimental group/condition, given as a discrete number and unit of measurement
☐	☒	A statement on whether measurements were taken from distinct samples or whether the same sample was measured repeatedly
☐	☒	The statistical test(s) used AND whether they are one- or two-sided<br>*Only common tests should be described solely by name; describe more complex techniques in the Methods section.*
☒	☐	A description of all covariates tested
☐	☒	A description of any assumptions or corrections, such as tests of normality and adjustment for multiple comparisons
☐	☒	A full description of the statistical parameters including central tendency (e.g. means) or other basic estimates (e.g. regression coefficient) AND variation (e.g. standard deviation) or associated estimates of uncertainty (e.g. confidence intervals)
☐	☒	For null hypothesis testing, the test statistic (e.g. *F*, *t*, *r*) with confidence intervals, effect sizes, degrees of freedom and *P* value noted<br>*Give P values as exact values whenever suitable.*
☒	☐	For Bayesian analysis, information on the choice of priors and Markov chain Monte Carlo settings
☒	☐	For hierarchical and complex designs, identification of the appropriate level for tests and full reporting of outcomes
☐	☒	Estimates of effect sizes (e.g. Cohen's *d*, Pearson's *r*), indicating how they were calculated

Our web collection on statistics for biologists contains articles on many of the points above.

Software and code

Policy information about availability of computer code

Data collection	No custom software or code was used for data collection.
Data analysis	Rnaseq - STAR Aligner (v. 2.5.1), RStudio (v. 3.5. 2 Eggshell Igloo), DESEQ2 (v. 3.12) Other Softwares for Analysis: GraphPad Prism 6 software (v. 9.0.1), Fiji Image J (v. 1.43)

For manuscripts utilizing custom algorithms or software that are central to the research but not yet described in published literature, software must be made available to editors and reviewers. We strongly encourage code deposition in a community repository (e.g. GitHub). See the Nature Research guidelines for submitting code & software for further information.

Data

Policy information about availability of data

All manuscripts must include a data availability statement. This statement should provide the following information, where applicable:
- Accession codes, unique identifiers, or web links for publicly available datasets
- A list of figures that have associated raw data
- A description of any restrictions on data availability

We are including the source data as an Excel file. The source data underlying figures 2b–e, 2g-j, 3b, 3d-j, 3m-o, 3p-q, 3r, 4b-i, 4k-l, 4n-p, 5b, 6a-b, 6d-g, supplementary figures 8a-h, 9a-i, 10a-b, 11a-f, 12, 13-a-b and uncropped Western blots are provided as a Source Data file. RNA-sequencing data that support the findings of this study are available in the Gene Expression Omnibus (GEO) database under accession number GSE168622.

Field-specific reporting

Please select the one below that is the best fit for your research. If you are not sure, read the appropriate sections before making your selection.

☒ Life sciences ☐ Behavioural & social sciences ☐ Ecological, evolutionary & environmental sciences

For a reference copy of the document with all sections, see nature.com/documents/nr-reporting-summary-flat.pdf

Life sciences study design

All studies must disclose on these points even when the disclosure is negative.

Sample size	Please see the information below with details about the sample size and statistics completed for each figure:<br>Figure 2:<br>(b,c) n≥30-40 cells from three independent differentiations, bar graph displaying one-way ANOVA- Bonferroni test when comparing GEMIN5 expression between H913R homozygous (H913RA6 and H913RA11) and heterozygous controls (H913RA21; ****<0.0001, p=ns, non-significant.<br>(d,e)) n=25-30 cells from three independent differentiations; bar graph displaying two tailed Mann-Whitney U test when comparing GEMIN5 expression between L1068P homozygous and heterozygous controls; ****<0.0001, p=ns, non-significant.<br>(g,h) n≥25 cells from three independent differentiations, two tailed Mann-Whitney U test; ****<0.0001, ***<0.001, p=ns, non-significant.<br>(i,j) n≥25 cells from three independent differentiations, two tailed Mann-Whitney U test; ****<0.0001, p=ns, non-significant.<br><br>Figure 3:<br>(b) n=4 independent biological replicates; two tailed Mann-Whitney U test; ***<0.001, **<0.01, *<0.05.<br>(d-j) n=4 independent biological replicates; one-way ANOVA- Bonferroni test; ****<0.0001, **<0.01, p=ns, non-significant.<br>(m-o) n=3 independent biological replicates; one-way ANOVA-Tukey test.<br>(p,q) n=6; two tailed Mann-Whitney U test; p=ns, non-significant.<br>(r) n=6; one-way ANOVA-Tukey test<br><br>Figure 4:<br>(b-i) n=5 independent biological replicates; one-way ANOVA- Bonferroni test; ****<0.0001,***<0.001, **<0.01, *<0.05, p=ns, non-significant.<br>(k) n=5 independent biological replicates; two tailed Mann-Whitney U test;***<0.001.<br>(i) n=5 independent experiments; two tailed Mann-Whitney U test;****<0.0001.<br>(m) n=12 flies; two tailed Mann-Whitney U test; *<0.05.<br>(o) n=25-39 flies; two tailed Mann-Whitney U test; ***<0.001.<br>(p) n=80 flies; Kaplan-Meier survival plot.<br><br>Figure 5:<br>b n=3 independent differentiations; two tailed Mann-Whitney U test;***<0.001, **<0.01.<br><br>Figure 6:<br>(a-g) n=3, Benjamini and Hochberg's approach; p-value and FDR value ≤0.05.
Data exclusions	No data was excluded from the study.
Replication	Each experiment was performed in biological replicates of 3-5 samples and was reproducible across each replicate.
Randomization	No randomization was used for any of the experiments in this manuscript. Randomization was not relevant to our studies, as we used cell culture systems and drosophila models; we did not use any clinical patient data that needed to be randomized.
Blinding	Double blind quantification methods were used for quantifications of Imunnofluorescence images and Drosophila experiments. The data was given to a third party member within the laboratory to eliminate any bias towards quantification methods.

Reporting for specific materials, systems and methods

We require information from authors about some types of materials, experimental systems and methods used in many studies. Here, indicate whether each material, system or method listed is relevant to your study. If you are not sure if a list item applies to your research, read the appropriate section before selecting a response.

Materials & experimental systems

n/a	Involved in the study	
	x	Antibodies
	x	Eukaryotic cell lines
x		Palaeontology and archaeology
x		Animals and other organisms
x		Human research participants
x		Clinical data
x		Dual use research of concern

Methods

n/a	Involved in the study	
x		ChIP-seq
x		Flow cytometry
	x	MRI-based neuroimaging

Antibodies

Antibodies used

For Immunofluorescence: : mouse anti-GEMIN5 (Millipore Sigma HPA037393, 1:1,000), mouse anti-GEMIN2 [2E17] (abcam ab6084, 1:500), mouse anti-GEMIN6/SIP2, (abcam ab88290, 1:500) rabbit anti-GEMIN4 (NOVUS Biologicals NB110-40591, 1:500), mouse anti-GEMIN3, clone 12H12 (Millipore Sigma 05-1533, 1:500), mouse anti-SMN (BD transduction 610646, 1:1,000), rabbit anti-U1A (NOVUS Biologicals NBP2-53095, 1:2,000), chicken anti- beta-III Tubulin (NOVUS Biologicals NB100-1612- 1:1,000), goat anti-MAP2 (Synaptic System-188 004, 1:1,000), and mouse anti-Ubiquitin. Alexa fluor-488, -568 and -647 secondary antibodies were used from Invitrogen.
For WB: mouse anti-tubulin (SIGMA, 1:10,000) anti-GEMIN5 (GenTex GTX130498, 1:1,000), mouse anti-GEMIN2 [2E17] (1:2,000), mouse anti-GEMIN6/SIP2 (1:5,000) rabbit anti-GEMIN4 (1:2,000), mouse anti-GEMIN3, clone 12H12 (1:1,000), mouse anti-SMN (1:5,000), and rabbit anti-U1A (NOVUS Biologicals NBP2-53095, 1:2,000).

Validation

All antibodies mentioned above with the catalog number and company information were validated by the company with proof of concept data on the website for both Western Blot and Immunofluorescence.

Eukaryotic cell lines

Policy information about cell lines

Cell line source(s)

The HEK293T and cells used were received fresh from ATCC and authenticated by their standard quality control procedures. The iPSC cell line sources are clearly listed in the methods section of the manuscript. D.escription of the passages used are provided in the materials and methods section

Authentication

The HEK293T cell lines were directly purchased from the ATCC and only low passage.
Routine testing of each individual iPSC clonal line were performed by the following methods: mycoplasma analysis, karyotype analysis was performed to ensure cells were free of abnormalities, and STR analysis was performed to validate the identity of the cells. DNA Sanger sequencing was also performed to validate the mutant GEMIN5 iPSC lines.

Mycoplasma contamination

The cell lines were negative for any mycoplasma contamination

Commonly misidentified lines
(See ICLAC register)

No commonly misidentified cell lines were used in the study.

Magnetic resonance imaging

Experimental design

Design type

Indicate task or resting state; event-related or block design.

Design specifications

MRI's on the patients were obtained by the clinical team at each site as part of their clinical care. MRI mages on sagittal and coronal T2 images of the cerebellum were reviewed and reports compiled for results of this study. Since this was a retrospective collection of clinical data, no standardized protocol was used in MRI acquisitio

Behavioral performance measures

State number and/or type of variables recorded (e.g. correct button press, response time) and what statistics were used to establish that the subjects were performing the task as expected (e.g. mean, range, and/or standard deviation across subjects).

Acquisition

Imaging type(s) — *Specify: functional, structural, diffusion, perfusion.*

Field strength — *Specify in Tesla*

Sequence & imaging parameters — *Specify the pulse sequence type (gradient echo, spin echo, etc.), imaging type (EPI, spiral, etc.), field of view, matrix size, slice thickness, orientation and TE/TR/flip angle.*

Area of acquisition — *State whether a whole brain scan was used OR define the area of acquisition, describing how the region was determined.*

Diffusion MRI ☐ Used ☐ Not used

Preprocessing

Preprocessing software — *Provide detail on software version and revision number and on specific parameters (model/functions, brain extraction, segmentation, smoothing kernel size, etc.).*

Normalization — *If data were normalized/standardized, describe the approach(es): specify linear or non-linear and define image types used for transformation OR indicate that data were not normalized and explain rationale for lack of normalization.*

Normalization template — *Describe the template used for normalization/transformation, specifying subject space or group standardized space (e.g. original Talairach, MNI305, ICBM152) OR indicate that the data were not normalized.*

Noise and artifact removal — *Describe your procedure(s) for artifact and structured noise removal, specifying motion parameters, tissue signals and physiological signals (heart rate, respiration).*

Volume censoring — *Define your software and/or method and criteria for volume censoring, and state the extent of such censoring.*

Statistical modeling & inference

Model type and settings — *Specify type (mass univariate, multivariate, RSA, predictive, etc.) and describe essential details of the model at the first and second levels (e.g. fixed, random or mixed effects; drift or auto-correlation).*

Effect(s) tested — *Define precise effect in terms of the task or stimulus conditions instead of psychological concepts and indicate whether ANOVA or factorial designs were used.*

Specify type of analysis: ☐ Whole brain ☐ ROI-based ☐ Both

Statistic type for inference (See Eklund et al. 2016) — *Specify voxel-wise or cluster-wise and report all relevant parameters for cluster-wise methods.*

Correction — *Describe the type of correction and how it is obtained for multiple comparisons (e.g. FWE, FDR, permutation or Monte Carlo).*

Models & analysis

n/a	Involved in the study
☒	☐ Functional and/or effective connectivity
☒	☐ Graph analysis
☒	☐ Multivariate modeling or predictive analysis
